# Characterization of PUD-1 and PUD-2, Two Proteins Up-Regulated in a Long-Lived *daf-2* Mutant

**DOI:** 10.1371/journal.pone.0067158

**Published:** 2013-06-14

**Authors:** Yue-He Ding, Yun-Guang Du, Shukun Luo, Yu-Xin Li, Tie-Mei Li, Sawako Yoshina, Xing Wang, Karsten Klage, Shohei Mitani, Keqiong Ye, Meng-Qiu Dong

**Affiliations:** 1 National Institute of Biological Sciences, Beijing, Beijing, China; 2 Graduate Program in Chinese Academy of Medical Sciences and Peking Union Medical College, Beijing, China; 3 Department of Physiology, Tokyo Women's Medical University, Tokyo, Japan; 4 Department of Cellular Biochemistry, Max Planck Institute of Biochemistry, Martinsried, Germany; Ohio State University, United States of America

## Abstract

*C. elegans* PUD-1 and PUD-2, two proteins up-regulated in *daf-2*(*loss-of-function*) (PUD), are homologous 17-kD proteins with a large abundance increase in long-lived *daf-2* mutant animals of reduced insulin signaling. In this study, we show that both PUD-1 and PUD-2 are abundantly expressed in the intestine and hypodermis, and form a heterodimer. We have solved their crystal structure to 1.9-Å resolution and found that both proteins adopt similar β-sandwich folds in the V-shaped dimer. In contrast, their homologs PUD-3, PUD-4, PUDL-1 and PUDL-2 are all monomeric proteins with distinct expression patterns in *C. elegans*. Thus, the PUD-1/PUD-2 heterodimer probably has a function distinct from their family members. Neither overexpression nor deletion of *pud-1* and *pud-2* affected the lifespan of WT or *daf-2* mutant animals, suggesting that their induction in *daf-2* worms does not contribute to longevity. Curiously, deletion of *pud-1* and *pud-2* was associated with a protective effect against paralysis induced by the amyloid β-peptide (1-42), which further enhanced the protection conferred by *daf-2*(*RNAi*) against Aβ.

## Introduction

The insulin/insulin-like signaling pathway (IIS) negatively regulates lifespan in a variety of species from *C. elegans*, *Drosophila* , to mice [1,2]. Mutant *C. elegans* carrying a single missense mutation in *daf-2*, which encodes the only insulin receptor in the worm, lives twice as long as the wild type (WT) [3]. The discovery that a single gene mutation can extend lifespan to such an extraordinary degree and that the lifespan regulation by IIS is conserved throughout evolution came as a surprise, because it was predicted that such genes would not exist based on a belief that the evolutionary pressure on any post-reproductive traits should be weak, if any [4]. While aging remains one of the last frontiers in biology, exceptionally long-lived mutants such as the *daf-2* worms inspire excitement—they are likely the key to understanding the molecular mechanisms of aging. Logically, one of the first steps is to determine the changes in gene expression in the *daf-2* mutants.

In *C. elegans*, most if not all insulin signaling transmits through a FOXO transcription factor called DAF-16 [1,2], thus, the downstream targets of DAF-16 are critical to the regulation of lifespan. A variety of experimental approaches have been applied to identifying DAF-16 targets, including microarrays, chromatin IP, quantitative mass spectrometry, and DamID [5–10].

We previously looked for DAF-16 targets by quantitative proteomics [8]. Among the 86 proteins that are up- or down-regulated in *daf-2*, some are well-known DAF-16 targets such as SOD-3, while others are more or less unexpected. Of the 86 proteins, F15E11.13 (encoded by two genes *F15E11*.*13* and *Y19D10B.7*) and F15E11.14 (also encoded by two genes *F15E11.1* and *F15E11*.14) are up-regulated the most (at least 4-fold). We have given these genes new names, *pud-1.1 (F15E11*.*13), pud-1.2* (*Y19D10B.7*), *pud-2.1* (*F15E11.1*), and *pud-2.2 (F15E11*.*14)*, in which *pud* stands for protein up-regulated in *daf-2*(*lf*). *pud-1.1* and *pud-1.2* are gene duplications with exactly the same DNA sequence, encoding the same protein PUD-1; and so are *pud-2.1* and *pud-2.2*, both encode PUD-2. They belong to an uncharacterized gene family, which we refer to as the PUD family. The other members of this gene family in *C. elegans* are *pud-3 (F15E11*.*15), pud-4 (F15E11*.*12), pudl-1* (*Y54G2A.46*), and *pudl-2* (*Y54G2A.47*), where *pudl* stands for *pud-like.*

A literature search finds that although these proteins or genes have not been characterized, they have appeared repeatedly in previous publications, often buried in the supplemental information as differentially expressed genes under various conditions. For example, a temperature shift of *glp-1*(*e2144ts*) mutant from 15 °C to 25 °C was accompanied with a remarkable increase of PUD-1 and PUD-2 [11]. A 7-fold increase of the PUD-2 protein level was detected in the *glp-4*(*bn2ts*) mutant compared to WT cultured at the same temperature of 25 °C [12]. Moreover, the PUD-1 protein was shown to build up conspicuously in transgenic *C. elegans* expressing a human 7-dehydrocholesterol reductase, an enzyme functioning in *de novo* biosynthesis of cholesterol [13]. Similarly, when the mRNA levels were measured, elevation of the *pud-1* and *pud-2* transcripts were ranked among the top eight in the *pept-1*(*lg601*) mutant [14], and among the top 15 in *rde-1*(*RNAi*) and *rde-4*(*RNAi*) mutants [15]. Decreased transcript levels of these genes were also observed, in *mdt-15*(*RNAi*) worms [16], in the *glp-4*(*bn2*)*; sek-1*(*ag1*) double mutant relative to the *glp-4*(*bn2*) single mutant [17], and in WT *C. elegans* exposed to high concentration of CO_2_ [18]. It is worth noting that the *glp-1* and *pept-1* mutants, in which increased expression of these genes have been detected, have a longevity phenotype similar to that of *daf-2*.

To understand the function of PUD-1 and PUD-2, and the biological significance of their up-regulation in *daf-2* mutants, we set out to analyze their expression patterns, overexpression phenotype, loss-of-function phenotype, interacting proteins, and protein structures.

## Materials and Methods

### Plasmids and *C. elegans* strains

The plasmids and the worm strains used in this study are listed in Tables S4 and S5 in File S1, respectively. Bristol N2 was used as the wild type strain. Worms were cultured on NGM plates at 20 °C unless indicated otherwise. Transgenic strains were constructed through microinjection of plasmids, and transgenes were chromosomally integrated by gamma-irradiation. Single copy insertion of transgenes was generated as described [19]. All of the integrated transgenic strains and the mutant strains were backcrossed to N2 at least four times.

### RNAi

RNAi by feeding was carried out at 20 °C as described [20]. For RNAi by injection, dsRNA was transcribed *in vitro* from linearized plasmid DNA using a kit (Promega), and the purified dsRNA was injected into the *C. elegans* gonad. The phenotype of the F1 progeny was analyzed.

### Quantitative RT-PCR

Total RNA was extracted from synchronized young adults using TRIZOL (Invitrogen), followed by the removal of contaminant DNA using DNase I. cDNAs were synthesized from the total RNA templates using a reverse transcription kit (Takara). Primers used for qPCR were oMD241[ACTTATCCGCATCCCATCCAGTGT]/oMD244 [ATGTCGA CTGATCCAACTCCACCA] for *pud-1*, oMD246[TGACAGAACAATGGGCCAAAGTGG]/ oMD247[GCCGGTATACTGATGAAGCACTTGGA] for *pud-2*, and actin-F[TGCCGCTCTT GTTGTAGACAATGG]/actin-R[TGACGTGGTCTTCCGACAATGGAT] for *actin* as the internal standard. qPCR was carried out on an ABI 7500 Fast real-time PCR system using a Takara realtime PCR kit (SYBR Premix Ex Taq^TM^ II).

### Mapping the boundaries of niDf209

The precise boundaries of *niDf209* and *niDf207* in JU258 were determined by nested PCR followed by sequencing. Primers pairs were designed in 1-kb intervals to cover the annotated flanking sequences (wormbase.org). *niDf209* is a 15,979-bp deletion with a 1,608-bp insertion. *niDf207* is a 4072-bp deletion.

### Generating hq5 and hq6

To help introduce *niDf209* (genetic position: -14.89) to the N2 background, *unc-60* (genetic position: -18.88) and *dpy-11* (genetic position: -0.02) were used to mark recombination events on Chr. V. JU258 was first crossed with DR181 *unc-60*(*m35*) *dpy-11*(*e224*), and DpynonUnc progeny with the recombination sites nearest to, and on the right side of *niDf209* were determined by PCR with oligos oMD307 and oMD308. Candidate alleles were crossed into DR35 *unc-60*(*m35*) and the resulting UncDpy progeny containing *niDf209* were selected for further validation. *hq6* was isolated using *niDf208* (16.7 Kb upstream of *niDf209*) as a marker of recombination to the left of *niDf209*. *hq5* was isolated using another marker upstream of *niDf208*. The linked *unc-60*(*m35*) and *dpy-11*(*e224*) alleles were removed through recombination after repeated backcrosses to N2.

### Lifespan assay

Unless indicated otherwise, all lifespan assays were carried out at 20 °C as described before [8]. Kaplan-Meier survival analyses were carried out using the SPSS software and the log-rank *p* values were reported.

### Heat tolerance assay

For each strain, 30 animals were shifted from 20 °C to 35 °C at the young adult stage and examined for survival every two hours.

### PA14 fast killing assay

The assay was performed as described previously [21].

### Paralysis assay

On day 0, 100 L4s were transferred to fresh NGM plates seeded with OP50 (10 worms/plate) and moved to new plates every two days. Paralyzed worms that could only move their heads were counted every day. Paralysis assay was terminated after 7 days to avoid mistaking old worm as “paralyzed”.

### Microscopy

All GFP and DIC images were taken using a Zeiss AxioImager M1 microscope.

### mRNA sequencing and data analysis

Total RNAs were extracted from the WT and *hq6* day-1 adults using TRIZOL (Invitrogen). The sequencing library was constructed by following the “mRNA sequencing sample preparation guide” provided by Illumina. The WT and *hq6* mRNA sequencing libraries were bar-coded and mixed in equal amounts. The paired-end RNA sequencing data were acquired on an Illumina GA II instrument (in two lanes). The paired-end RNA-seq 36 base pair reads were mapped to the *C. elegans* genome (WS220) using the TopHat software, allowing no more than two mismatches per sequencing read. In TopHat mapping, the expected (mean) inner distance between mate pairs was set to 100 bp, the standard deviation for the distribution on inner distances between mate pairs was set to 50 bp. Only uniquely mapped paired-end reads were extracted for subsequent analysis. Differential gene expression analysis was performed with the bioconductor package DESeq. Genes with reads number ≤ 10 in both samples were not included in the DESeq analysis. In total, the two samples produced 18 million uniquely mapped paired-end sequencing reads (9 million per sample). In a separate experiment, single-end RNA sequencing data were acquired for three independent WT N2 samples on Illumina HiSeq 2000.

### Immunoprecipitation and Mass Spec analysis

A single-copy insertion strain expressing FLAG-tagged PUD-1 and the control N2 strain were cultured on High-growth (HG) plates and harvested for anti-FLAG IP. Proteins bound to the M2 beads (Sigma) were eluted using 0.5µg/µl 3xFLAG peptides. SDS-PAGE analysis followed by silver staining revealed three protein bands in the FLAG::PUD-1 IP but not in the control IP. These bands were cut from the gel, digested with trypsin, and subjected to LC-MS/MS analysis on LTQ-orbitrap (Thermo, Fisher). Proteins were identified by searching the MS/MS spectra against a *C. elegans* protein database WS217 using Prolucid and filtering the search results with DTASelect 2.0.

### Protein purification

The PUD family proteins were expressed in the *Escherichia coli* BL21(DE3) strain (Novagen) induced with 0.2 mM isopropyl β-D-1-thiogalactopyranoside normally for 8 h at 25 °C. PUDL-1 and PUDL-2 were expressed at 16 °C for 16 h. PUD-1 and PUD-2 were coexpressed and copurified as a dimer. The harvested cells were resuspended in buffer P500 (50 mM phosphate pH 7.6, 500 mM NaCl) and lysed using a high pressure cell disruptor (JNBIO) followed by sonication. The cell lysates were clarified by centrifugation and passage through a 0.45-μm filter and loaded onto a 5-ml HisTrap column (GE healthcare). The column was washed by 50 mM imidazole in P500. The protein was eluted with 500 mM imidazole in P500 and incubated with ULP1 protease to cleave the His-SMT3 tag. After concentrating and dilution with 20 mM HEPES-K, pH 7.6, the sample was passed through a HisTrap column to remove the cleaved tag and uncleaved protein. The PUD-1/PUD-2 complex was further purified with a Superdex 200 column (GE healthcare) equilibrated in buffer E250 consisting of 5 mM HEPES-K, pH 7.6 and 250 mM KCl. The PUD-1/PUD-2 complex was labeled with seleno-methionine by inhibiting the methionine biosynthesis pathway and purified with the same procedure as unlabeled complex. The protein was concentrated by Amicon Ultra-15 units and stored at -80 °C.

### Crystallization and structure determination

The Se-labeled full-length PUD-1/PUD-2 complex was crystallized by mixing 1 μl of protein sample (46 mg/ml in E250) with 1 μl of well solution consisting of 0.2 M NH_4_H_2_PO_4_, 0.1 M Tris-HCl pH 8.5 and 40% 2-methyl-1,3 propanediol using the hanging drop vapor diffusion method at 20 °C. The crystal was flash frozen in liquid nitrogen without further cryoprotection. The diffraction data were collected at the wavelength of selenium peak at Shanghai Synchrotron Radiation Facility (SSRF) beamline BL17U and processed by HKL2000 [22]. The structure was solved by single-wavelength anomalous diffraction (SAD) method using a 3.6-Å dataset. The heavy atoms were located by SHELXD [23] and the phase calculation and density modification were conducted with SHARP [24]. The crystal belongs to space group P3 _2_21 and contains two copies of PUD-1/PUD-2 heterodimer in the asymmetric unit. The structural model was built in COOT [25] and refined with Refmac to a free R-factor of 0.38 at 3.6 Å resolution [26].

The PUD-1 (9-151) and PUD-2 (7-152) complex (35 mg/ml in E250) was crystallized in 0.1 M HEPES-Na pH 8.5 and 4.3 M NaCl. The crystal was cryoprotected with 10% glycerol in the mother solution and flash frozen in liquid nitrogen. A dataset was collected at SSRF-BL17U to 1.9 Å resolution. The structure was solved by molecular replacement in PHASER using the initial heterodimeric structure of full-length complex as a search model [27]. The crystal belongs to space group P2_1_2_1_2_1_ and contains one heterodimer in the asymmetric unit. The final model was refined with Refmac to 1.9 Å resolution and contains residues 9-151 of PUD-1, an N-terminal serine residue from the expression vector of PUD-1, residues 7-87 and 91-152 of PUD-2, one glycerol molecule, 10 chlorine ions and 326 waters. Analysis with RAMPAGE shows that 97.9% of the residues are in most favored regions and 2.1% in allowed regions [28]. Structural figures were prepared in PyMOL [29].

### Size exclusion chromatography

All six *C. elegans* proteins of the PUD protein family were individually purified through one-step HisTrap chromatography with the His-SMT3 tag uncleaved. Each protein (500 μg) was loaded individually or in combination in a total of 200 μl volume to a Superdex-200 10/300 GL column (GE healthcare) running in buffer E250.

## Results

### The PUD-1 and PUD-2 protein abundance increase in *daf-*2 is independent of *daf-16*

The protein levels of PUD-1 and PUD-2 are greatly elevated in *daf-2* mutants [8]. To find out whether this is due to activated gene expression by DAF-16, the key transcription factor normally inhibited by signaling from DAF-2 in WT *C. elegans*, we quantified their mRNA levels in WT, *daf-2*, *daf-16*, and *daf-16; daf-2* double mutants by quantitative PCR. The *pud-1* and *pud-2* mRNA levels increased in the *daf-2*(*e1370*) mutant relative to the WT, and this moderate increase required *daf-16* (Figure S1a in File S1). Interestingly, at the protein level both the *daf-2* mutant and the *daf-16; daf-2* double mutant had higher amounts of PUD-1 and PUD-2 than WT (Figure S1b-c in File S1), even though the mRNA templates of the two proteins were reduced in the double mutant. We thus propose that these proteins are up-regulated in the *daf-2* mutant mainly through a post-transcriptional mechanism independent of DAF-16.

### Overexpression of PUD-1 and PUD-2 did not extend lifespan

**Figure 1:**
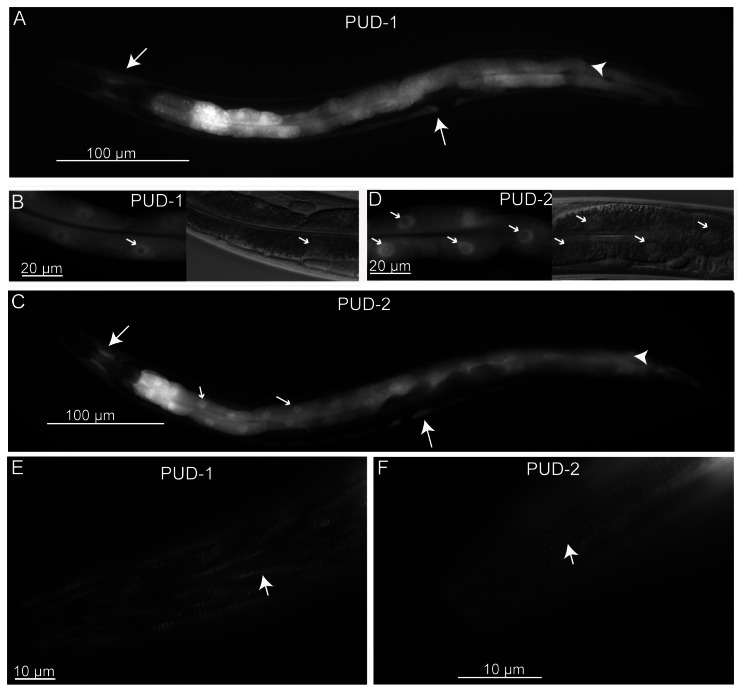
Expression of *P*_*pud-1*_ *::GFP::pud-1* and *P*_*pud-2*_ *::GFP::pud-2* from single-copy transgenes inserted into the *C. elegans* genome. PUD-1 (**A**, **B**, **E**) and PUD-2 (**C**, **D**, **F**) are both expressed the intestine (arrowheads) and hypodermis (long arrow). In the intestinal cells, PUD-1 (**B**) and PUD-2 (**D**) are distributed diffusely in the cytoplasm and the nucleoplasm, and largely excluded from the nucleolus (short arrow) expect for one or more nuclear puncta. The nucleolar GFP puncta are more distinct in high-copy transgenic strains (Figure S1). PUD-1 (**E**) and PUD-2 (**F**) are both localized in the hypodermal fibrous organelles with a characteristic circumferential orientation.

**Figure 2:**
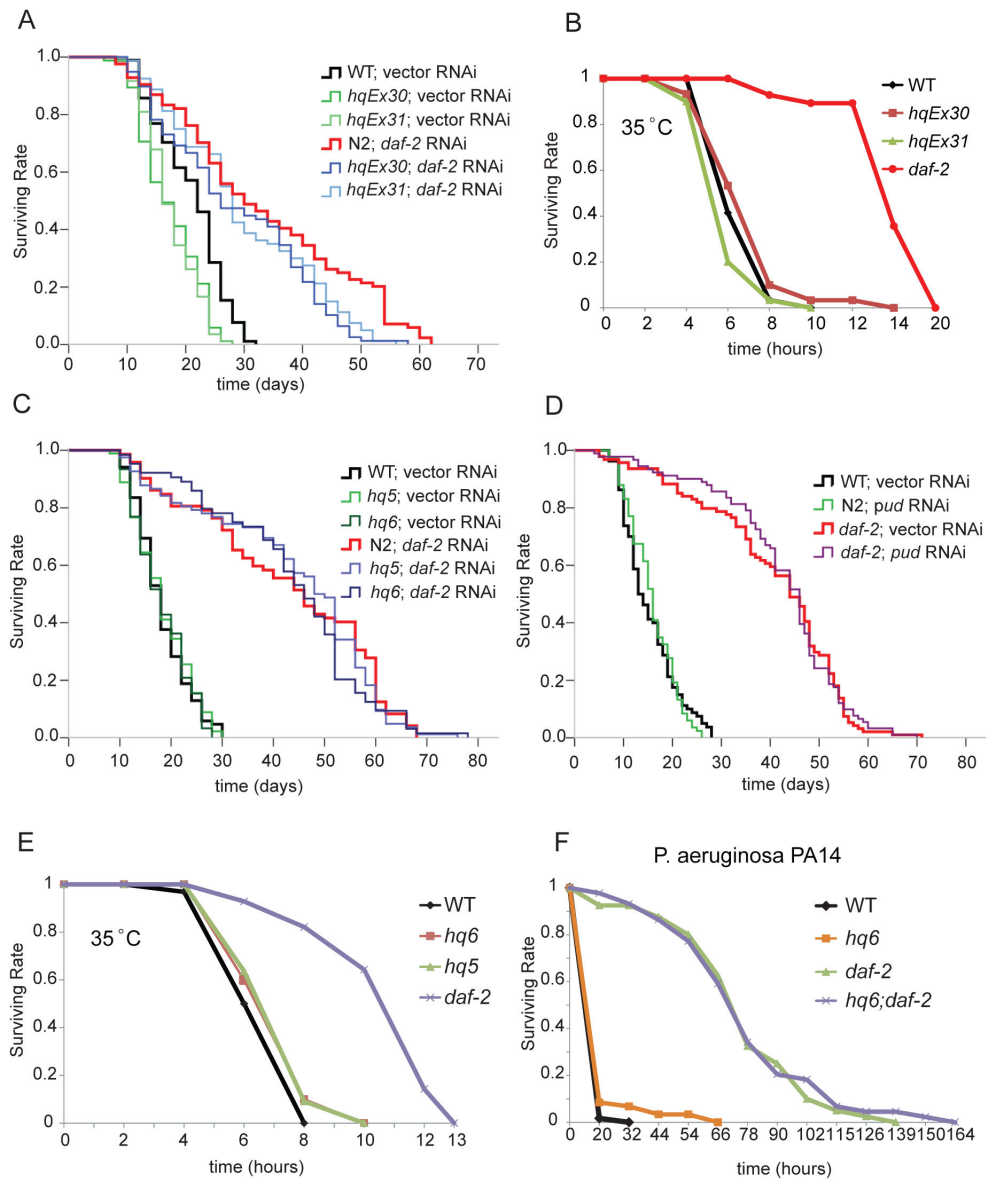
Neither overexpression nor deletion of *pud-1* and *pud-2* extended lifespan in WT or *daf-2* animals. (**A**) *hqEx30* and *hqEx31*, two extra-chromosomal transgenes expressing untagged PUD-1 and PUD-2, slightly shortened lifespan of WT and *daf-2*(*RNAi*) worms (*p* < 0.014 between *hqEx30* or *hqEx31* and WT with or without *daf-2* RNAi, N>77, 20 °C). (**B**) *hqEx30* and *hqEx31* animals were as sensitive to thermal stress as the WT. Stress resistant *daf-2*(*e1370*) served as a positive control (35 °C, N>27). (**C**) *hq5* and *hq6*, two deletion alleles that removed all the gene copies of *pud-1* and *pud-2*, did not affect the lifespan of WT or *daf-2*(*RNAi*) animals (N>63). (**D**) Knocking down *pud-1* and *pud-2* by heritable RNAi (via injection of dsRNA into parent worms) had no effect on WT or *daf-2*(*e1370*) lifespan (N>79). (**E**-**F**) *hq5* and *hq6* did not affect the survival of *C. elegans* at 35 °C (**E**) (N>27, representative of two experiments) or the pathogenic bacteria P. aeruginosa PA14 (**F**) (N >39, representative of three repeats).

To find out if the increased PUD-1 and PUD-2 protein amounts contribute to the longevity of the *daf-2* mutant, we overexpressed these proteins in WT animals to mimic their induction in *daf-2*. We generated two overexpression constructs, pYG2 and pWX1, in which the genomic sequences of *pud-1* and *pud-2* were fused to the GFP coding sequence, respectively (Figure S2a in File S1). Because *pud-1* and *pud-2* are adjacent genes transcribed from the same intergenic promoter region in opposite directions, un-tagged PUD-2 and a PUD-1::GFP fusion protein were expressed from the pYG2 transgene arrays *hqIs24* and *hqIs28*, while un-tagged PUD-1 and PUD-2::GFP were expressed from the pWX1 transgene array *hqIs60*. PUD-1::GFP and PUD-2::GFP are both expressed in the intestine and hypodermis, from either the multi-copy (Figure S2 in File S1) or the single-copy transgenes (Figure 1). None of the high-copy arrays altered the WT lifespan. Perplexingly, *hqIs28* and *hqIs60*, but not *hqIs24*, further extended the lifespan of *daf-2*(*e1370*) and *daf-2*(*RNAi*) animals by 27-38% (Figure S3 in File S1), and *hqIs60* animals also showed increased resistance to thermal stress (not shown). To clarify the overexpression phenotype, we made four additional transgene arrays (*hqEx30, hqEx31*, *hqEx50*, and *hqEx52*) from which untagged PUD-1 and untagged PUD-2 were co-expressed. None of them extended the *C. elegans* lifespan in either the WT or *daf-2*(*RNAi*) background; *hqEx30, hqEx31*, and *hqEx52* even shortened lifespan somewhat (Figure 2A and data not shown). *hqEx52* and another transgene *hqEx47* that was made by co-injection of *HA::pud-1::GFP* and *HA::pud-2::Cherry* under their native promoters failed to alter the lifespan of *daf-2*(*RNAi*) or WT worms (not shown). We also examined, *hqEx30, hqEx31*, and *hqEx47* animals for resistance to thermal stress at 35 °C and found that they were not different from WT worms (Figure 2B and data not shown). We thus conclude that overexpression of PUD-1 and PUD-2 does not extend the lifespan of WT or *daf-2* animals. We suspect that the longevity phenotype associated with *hqIs60* and *hqIs28* may be due to independent background mutations linked to the transgenes. Because the transgenes were integrated into different chromosomes and each was backcrossed to WT at least four times, the chance for two mutations at different loci to cause the same phenotype should be extremely rare, but it appeared to be what had occurred in this case.

### Deletion of *pud-1* and *pud-2* did not shorten the *daf-2* lifespan 

We next asked whether the longevity phenotype of the *daf-2* mutant requires the gene activities of *pud-1* and *pud-2*. Since knocking down of these genes using the RNAi-by-feeding method [20], either individually or in combination, failed to effectively reduce the fluorescence intensity of PUD-1::GFP or PUD-2::GFP in transgenic strains, we set out to obtain deletion alleles. Six members of the PUD gene family in *C. elegans*–*pud-1.2, pud-2.2, pud-3, pud-4, pud-1.1 and pud-2*.*1-*are next to each other on Chromosome V (Figure S4a in File S1) and the other two–*pudl-1* and *pudl-2*–are adjacent genes on Chromosome IV (Figure S4b in File S1). Fortunately, the six-gene cluster on Chromosome V is deleted in JU258, a wild *C. elegans* strain isolated from the island of Madeira. We mapped the precise boundaries of this deletion, *niDf209*, and found that in addition to the PUD gene cluster, three flanking genes (*Y19D10B.5, Y19D10B.6* and *F15E11*.11) are also deleted in *niDf209* (Figure S4a in File S1). The three flanking genes each have at least one paralog in the *C. elegans* genome with 40% to 90% sequence similarity, so the deletion of them likely has little phenotypic consequence. Thus far, no phenotype of any kind has been associated with any of the three genes.

We transplanted *niDf209* from JU258 to the standard WT N2 background by homologous recombination (Figure S5 in File S1). Briefly, JU258 was crossed to *unc-60*(*m35*) *dpy-11*(*e224*) (N2 background). The nonUnc *Df* Dpy worms descended from the first cross were mated with *unc-60*(*m35*) (N2 background), and the resulting Unc *Df* Dpy recombinants were selected and backcrossed to N2 repeatedly until nonUnc *Df* nonDpy recombinants were obtained, and these recombinants were further backcrossed to N2 for five more times. We obtained two alleles, *hq5* and *hq6*, that harbor *niDf209* in the N2 background. *hq5* has two deficiencies–*niDf208* and *niDf209*–from JU258 whereas *hq6* has only *niDf209* (Figure S4a in File S1); the latter was used in most of the deletion phenotype analyses.

Both *hq5* and *hq6* are morphologically normal, their locomotion and egg production are also WT-like (data not shown). We found that neither *hq5* nor *hq6* affected the lifespan of WT or *daf-2*(*RNAi*) animals (Figure 2C). We also made a composite RNAi construct pYG17 in which 222-323 nucleotide sequences each targeting one or two PUD gene family members were concatenated together (Figure S6 in File S1). Injection of the dsRNA transcribed *in vitro* from pYG17 into *C. elegans* (*pud* RNAi) reduced PUD-1::GFP and PUD-2::GFP levels by at least 50% in the next generation (data not shown). Consistent with the deletion alleles, *pud* RNAi did not change the lifespan of WT or *daf-2*(*e1370*) worms (Figure 2D).

### *hq6* conferred resistance to Aβ toxicity 

**Figure 3:**
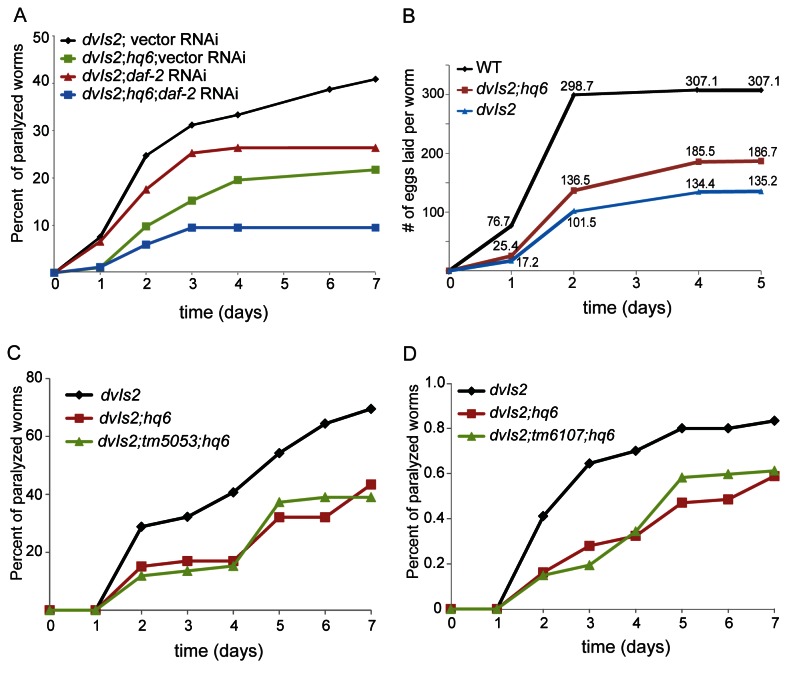
*hq6* animals are more resistant to Aβ(1-42) toxicity. (**A**) In either WT or *daf-2*(*RNAi*) background, *hq6* conferred resistance to paralysis induced by *dvIs2*, which expressed Aβ(1-42) in the body wall muscles (N ≥83, representative of three experiments). (**B**) *hq6* increased the brood size of *dvIs2* worms (N ≥10, representative of three experiments). (**C**-**D**) *tm6107;hq6* and *tm5053; hq6* double mutants showed similar resistance to Aβ toxicity as the *hq6* single mutant.

Since *daf-2* and many other IIS mutants are not only long-lived, but also resistant to various types of stress such as high temperature, pathogen, and Aβ toxicity [30-32], we asked if the deletion of *pud-1* and *pud-2* would affect stress tolerance. We found that the survival curves of *hq5* and *hq6* animals were similar to that of WT at 35 °C (Figure 2E). Likewise, in a fast killing assay using PA14, a pathogenic *P. aeruginosa* strain, we did not observe consistent difference between WT and *hq6*, or between *daf-2* and *daf-2; hq6* (Figure 2F). However, the *hq6* allele protected WT animals from paralysis induced by Aβ(1-42) expressed in the body-wall muscles [33], and further enhanced the resistance of *daf-2*(*RNAi*) animals to the Aβ toxicity (Figure 3A). It also improved the brood size of Aβ transgenic worms (Figure 3B). The fact that *hq6 and daf-2*(*RNAi*) had a synergistic effect on Aβ resistance and that *hq6* did not shorten the *daf-2* lifespan both argue for the point that PUD-1 and PUD-2 are not connected to the longevity of *daf-2* animals.

In addition to *hq5* and *hq6*, we also isolated *tm5053* and *tm6107* on chromosome IV. In *tm5053*, part of the coding sequence of *pudl-1* and much of the intergenic region between *pudl-1* and *pudl-2* are deleted. In *tm6107*, both *pudl-1* and *pudl-2* are deleted (Figure S4b in File S1). No obvious defects were detected in *tm5053* or *tm6107* worms. The *tm6107;hq6* and *tm5053; hq6* double mutants were as resistant to Aβ toxicity as the *hq6* single mutant (Figure 3C-D).

### The PUD family members are likely positive regulator of a subset of collagen genes and a subset of sperm protein genes

To find out the functions of *pud-1* and *pud-2* in a non-biased way, we compared the gene expression profiles of the WT and *hq6* animals using next-generation mRNA sequencing. At 5% false discovery rate and excluding the genes deleted in the *hq6* allele, only 21 genes were differentially expressed in *hq6* animals compared with the WT (Table 1), and 19 of them were down-regulated in *hq6*. Most of these genes had a modest mRNA abundance change in *hq6* except for *haf-6* (2.5-fold decrease) and *srbc-15* (no transcripts detected). Significantly enriched in this short list of genes are the ones encoding collagens (*col-39, col-125, col-133, col-147, col-149*, and *col-179*) or sperm proteins (a major sperm protein gene *msp-77*, sperm specific family class P or Q genes *ssp-34, ssq-1, ssq-2*, and *ssq-4*) (*p* < 0.001 for the enrichment of either group of genes, hypergeometric test). The mRNA-seq data suggest that one or more of the genes deleted in *hq6* are positive regulators of collagen or sperm protein production.

**Table 1:** Differentially expressed genes in*hq6* compared to wild type.

**Gene Name**	**Fold Change (*hq6vs* N2)**	**Adjusted P-value**	**Gene Description (Concise)**
*col-133*	0.37	1.0E-07	Collagens (type IV and type XIII), and related proteins
*col-147*	0.30	1.0E-07	Collagens (type IV and type XIII), and related proteins
*haf-6*	0.17	2.1E-06	A half-molecule ATP-binding cassette (ABC) transporter
*col-39*	0.37	3.8E-05	Collagens (type IV and type XIII), and related proteins
*ssp-34*	0.45	2.1E-04	Sperm Specific family, class P
*C15C6.2*	0.39	2.6E-04	Unnamed protein
*ssq-1*	0.55	3.3E-04	Sperm-Specific family, class Q
*col-149*	0.49	4.5E-04	Collagens (type IV and type XIII), and related proteins
*gipc-2*	0.49	2.4E-03	GIPC (RGS-GAIP Interacting Protein C) homolog
*col-179*	1.69	5.7E-03	Collagens (type IV and type XIII), and related proteins
*F08H9.2*	0.48	5.7E-03	Unnamed protein
*nspd-7*	0.53	5.7E-03	Nematode Specific Peptide family, group D
*nspd-1*	0.57	6.1E-03	Nematode Specific Peptide family, group D
*ssq-2*	0.59	7.0E-03	Sperm-Specific family, class Q
*rpl-39*	1.57	1.2E-02	Large ribosomal subunit L39 protein
*Y57G11A.2*	0.46	1.4E-02	Unnamed protein
*Y45F10C.4*	0.50	1.9E-02	Unnamed protein
*srbc-15*	0.00	4.3E-05	Serpentine Receptor, class BC (class B-like)
*col-125*	0.32	2.4E-02	Collagens (type IV and type XIII), and related proteins
*msp-77*	0.54	4.0E-02	Major Sperm Proteins (MSPs)
*ssq-4*	0.61	4.7E-02	Sperm-Specific family, class Q

Mutations of collagen genes have been associated with morphology or locomotion phenotypes such as Dpy (dumpy), Lon (long), Bli (blister), or Rol (roller) [34]. However, *hq6* animals appear to be normal in morphology and movement, so the reduced expression of the collagen genes appears to be inconsequential. This is reminiscent of *rol-6*, of which the null mutants are essentially WT-like while the missense mutations cause a strong roller phenotype [35]. Alternatively, the reduced expression of five collagen genes might be compensated by the up-regulation of *col-179* (Table 1). No gross defects were detected in the egg-laying behavior or the brood size of *hq6* animals, suggesting that *hq6* sperm are functional despite a reduction in the mRNA levels of five sperm proteins.

### PUD-1 and PUD-2 form a heterodimer *in vitro* and *in vivo*

**Figure 4:**
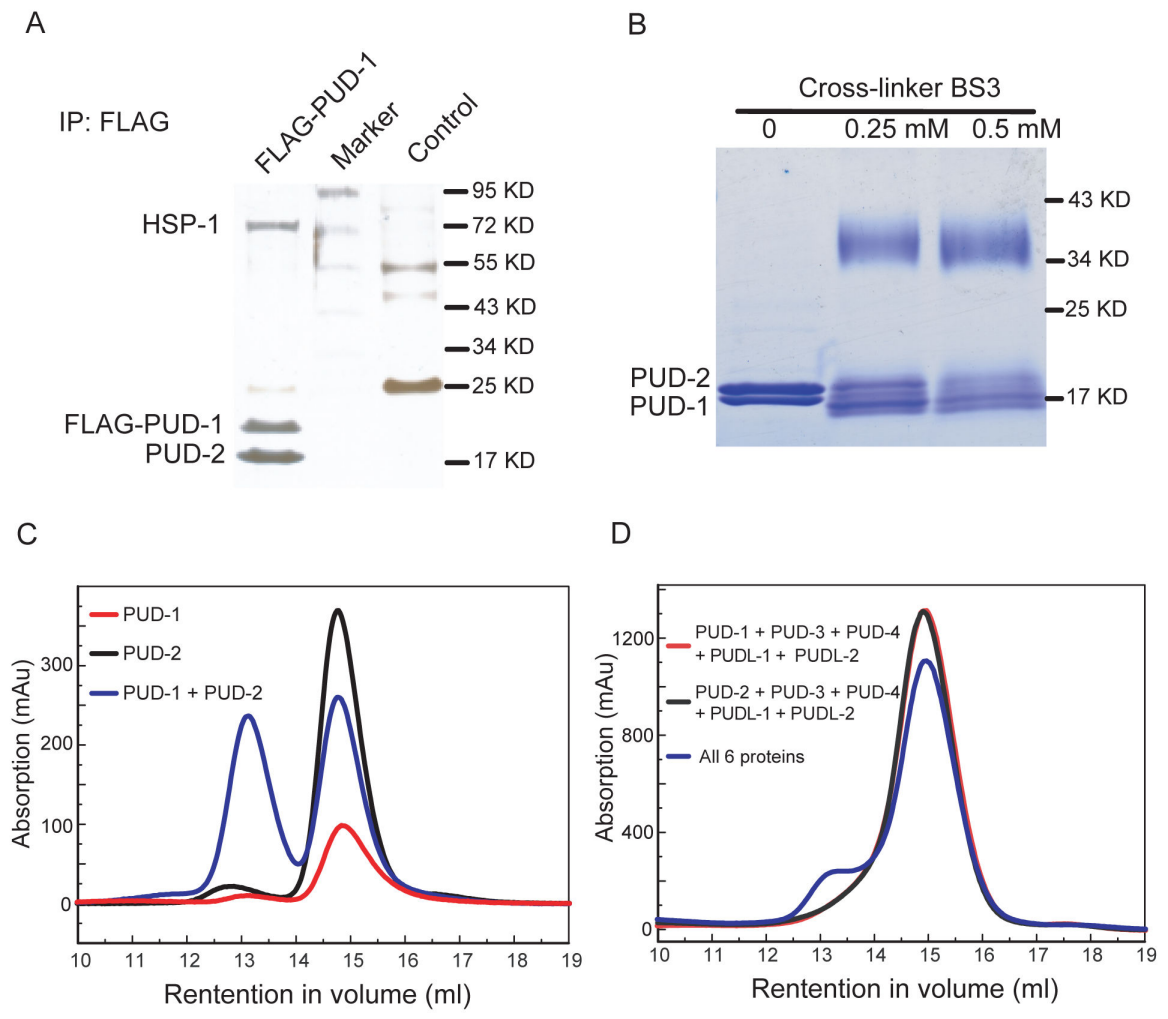
PUD-1 and PUD-2 form a heterodimer. (**A**) FLAG IP followed by MS analysis identified HSP-1 and PUD-2 as binding proteins associated with FLAG-PUD-1. Shown is the silver stained gel of the IP products before MS analysis. (**B**) Purified recombinant PUD-1 and PUD-2 can be cross-linked together and the cross-linked species seems to be a heterodimer. (**C**) The PUD-1 and PUD-2 heterodimer, each with a SMT3 tag, was also observed by gel filtration. (**D**) Gel filtration analysis of the *C. elegans* PUD gene family proteins. SMT3 tagged proteins were loaded onto a Superdex-200 10/300 column individually or in indicated combinations.

We also tried to infer the functions of PUD-1 and PUD-2 from their binding proteins. Using the MosSCI technique [19], we generated a single copy transgene expressing FLAG-tagged PUD-1 under its native promoter (*P*_*pud-1*_ *::FLAG::pud-1*). Using an anti-FLAG antibody, we immunoprecipitated (IP) FLAG::PUD-1 and analyzed the protein bands in the IP by mass spectrometry. Endogenous PUD-2 and HSP-1, a constitutively expressed Hsc70 protein that is also inducible upon heat shock [36], were found associated with FLAG::PUD-1 (Figure 4A). The intensity of the silver-stained protein bands suggested that PUD-1 and PUD-2 have a 1:1 stoichiometry, while HSP-1 is substoichiometric. *In vitro* cross-linking and gel-filtration experiments using purified recombinant PUD-1 and PUD-2 indicated that they form a heterodimer (Figure 4B–D). With this information, we revisited the protein and mRNA quantitation results and realized that indeed, they had similar fold-change values in the mutants relative to the WT (Figure S1 in File S1 and [8]), and their expression patterns were the same (Figure 1 and Figure S2 in File S1). Together, our results demonstrate that PUD-1 and PUD-2 form a heterodimer *in vivo* and suggest that their expressions are regulated as a single unit.

The association of PUD-1 and PUD-2 with HSP-1 is intriguing. Although no sequence homology can be found between the PUD family proteins and any heat shock proteins, PUD-1 and PUD-2 appear to be regulated like heat shock proteins. For example, they are markedly induced by an increase of temperature from 20 °C to 27 °C (Figure S2d-e in File S1) or by exposure to cadmium [37]. Moreover, from an analysis of genes differentially regulated by the *C. elegans* RB protein LIN-35, a non-E2F binding element was found in the promoter regions of the small heat shock protein genes, and it was also found in the promoters of *pud-1* and *pud-2* [38]. We tested if the purified PUD-1/PUD-2 heterodimer displayed any chaperone activity in an *in vitro* assay [39], and found that it did not (data not shown). There is a possibility that this heterodimer may act as a co-chaperone of HSP-1, but more likely is a substrate of HSP-1.

### The crystal structure of the PUD-1 and PUD-2 heterodimer

**Figure 5:**
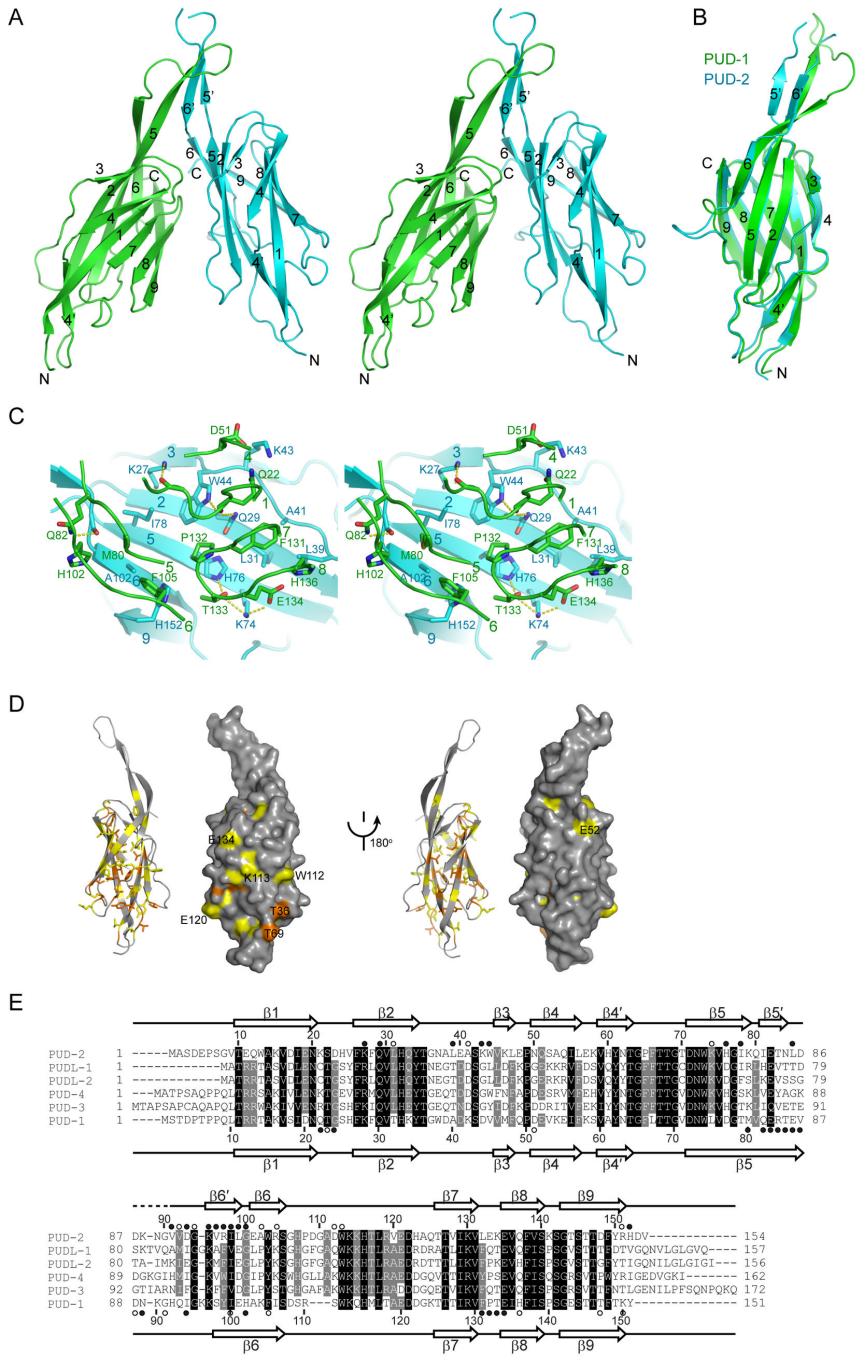
Structure of the PUD-1 and PUD-2 heterodimer. (**A**) Ribbon representation of the PUD-1 and PUD-2 heterodimer in cross-eye stereoview. PUD-1 is green and PUD-2 is cyan. The β-strands are labeled with numbers and the N and C termini are indicated. (**B**) Structural superposition of PUD-1 and PUD-2 subunit. (**C**) Interactions at the dimer interface. For clarity, only residues at the interface are shown for PUD-1. Oxygen is red, nitrogen is blue, sulfur is orange and carbon is green for PUD-1 and cyan for PUD-2. Hydrogen bonds are shown as yellow dashed lines. (**D**) Conserved surface. The ribbon and surface representations of PUD-1 structure are shown side-by-side and in two opposite orientations. The residues conserved in 100% and 80% of the 31 homologs of PUD-1 and PUD-2, as shown in Figure S7, are colored orange and yellow, respectively, for side chain atoms. (**E**) Sequence alignment of the PUD family proteins in *C. elegans*. Residues with 100% and 80% conservation are shaded with black and grey, respectively. The secondary structures observed in the crystal structure are shown on the top for PUD-2 and at the bottom for PUD-1. Residues whose surface area is buried by 10-30 Å^2^ and at least 30 Å^2^ upon dimerization are denoted by open and solid circles, respectively.

Next, we asked if these proteins have structural homologs that could give us clues about their function. The structure of the PUD-1/PUD-2 complex was first determined at 3.6 Å resolution for full length proteins by Se-phasing. The initial model shows that a few terminal residues are disordered. We then prepared a fragment of PUD-1 with residues 9-151 and a fragment of PUD-2 with residues 7-152 and obtained high quality crystals for the slightly truncated complex. The structure of the truncated complex was determined to 1.9 Å resolution with an R_work_/R_free_ of 0.192/0.225 and is discussed below (Figure 5, Table S1 in File S1).

In the structure, both PUD-1 and PUD-2 adopt similar β-sandwich folds that further associate with each other into a heterodimer (Figure 5A). As expected from 54% sequence similarity between PUD-1 and PUD-2, the two subunit structures can be well superimposed with a root mean square deviation (RMSD) of 1.277 Å over 118 Cα pairs (Figure 5B).

The β-sandwich fold of PUD-1 and PUD-2 is composed of 9 major β-strands arranged into two sheets. One sheet is formed by strands β3, β2, β5, β6 and β9 in an antiparallel manner, whereas the other sheet is composed of strands β4, its extension β4', β1, β7, β8 and β9 in a mixed manner, where all adjacent strands are aligned in antiparallel except β1 and β7 that are aligned in parallel. The strand β9 pairs with both sheets and closes one side of the sandwich. In addition, strands β5 and β6 project out from the sandwich body and form a prominent protrusion involved in dimerization.

The sandwich structures of PUD-1 and PUD-2 contact each other in a face-to-back manner with an inclination angle of 30^°^, forming a V-shaped dimer. The dimerization buries 1081 Å^2^ of solvent accessible surface area per subunit and is mediated by two interfaces. In the first interface, one end of the PUD-1 sandwich, which is composed of the β7-β8 loop, the β1-β2 loop and the exposed regions of strands β5 and β6, packs against the β3-β9 sheet of PUD-2 (Figure 5C). This interface is stabilized by hydrophobic and polar interactions. The hydrophobic interactions involve residues M80, F105, F131, P132, H136 of PUD-1 and residues L31, L39, A41, W44, H76, I78, A102 and H152 of PUD-2. A number of polar and water-mediated interactions are present at more peripheral regions. The second dimer interface is constituted by the extensions of β5 and β6 of both PUD-1 and PUD-2 (named β5' and β6' in PUD-2). They form an intermolecular antiparallel 4-stranded sheet with PUD-1 β5 aligned with PUD-2 β6' (Figure 5A.) 

Despite strong similarity between PUD-1 and PUD-2 structures, the dimer interface is asymmetric and engages nonequivalent faces of each subunit. Sequence alignment shows the residues at the dimer interface are dissimilar in PUD-1 and PUD-2, which accounts for the specificity of the dimerization mode (Figure 5E).

### Functional implication of the PUD-1/PUD-2 structure

A BLAST search in a non-redundant protein database of NCBI shows that PUD-1 and PUD-2 homologs have a very limited distribution across species and can only be identified in three worm species, five fungi and two bacteria (Figure S7 in File S1). Mapping the residues conserved in these sequences on the PUD-1 structure reveals a conserved surface patch at one end of the β-sandwich, opposite to the dimerization end (Figure 5D). The patch is mainly composed of residues from β6-β7 loop, β4’-β5 loop and the N-terminal part of β2-β3 loop. This region is likely important to the function of the PUD family proteins.

A DALI search shows that the β-sandwich of PUD-1/PUD-2 bears some topology similarity with proteins of diverse function, such as pore-forming cytolysin, transporter transthyretin and chaperone FaeE involved in pilus assembly (Z score to 6.9). However, the functional insight provided by these structural homologs is vague.

To summarize, after the genetic, genomic, biochemical, and structural analyses described above, much has been learned about the PUD-1 and PUD-2 proteins, but the significance of their biological function remains unclear.

### Characterization of other *C*. *elegans* homologs of PUD-1 and PUD-2

We determined the expression patterns of all the PUD gene family members in *C. elegans* by GFP fusion proteins, and the result is summarized in Table S2 in File S1. As described above, *pud-1* and *pud-2* are expressed strongly and uniformly in the intestine, and less strongly in the hypodermis (Figure 1 and Figure S2 in File S1). Besides, their GFP fusion proteins were both seen in fibrous organelles, the hypodermal hemi-desmosome structures that fasten muscles to the cuticle (Figure 1E-F). At the subcellular level, both proteins were found distributed diffusely in the cytoplasm and the nucleoplasm, and largely excluded from the nucleolus except for one or more nucleolar puncta (Figure S2b-c in File S1).

**Figure 6:**
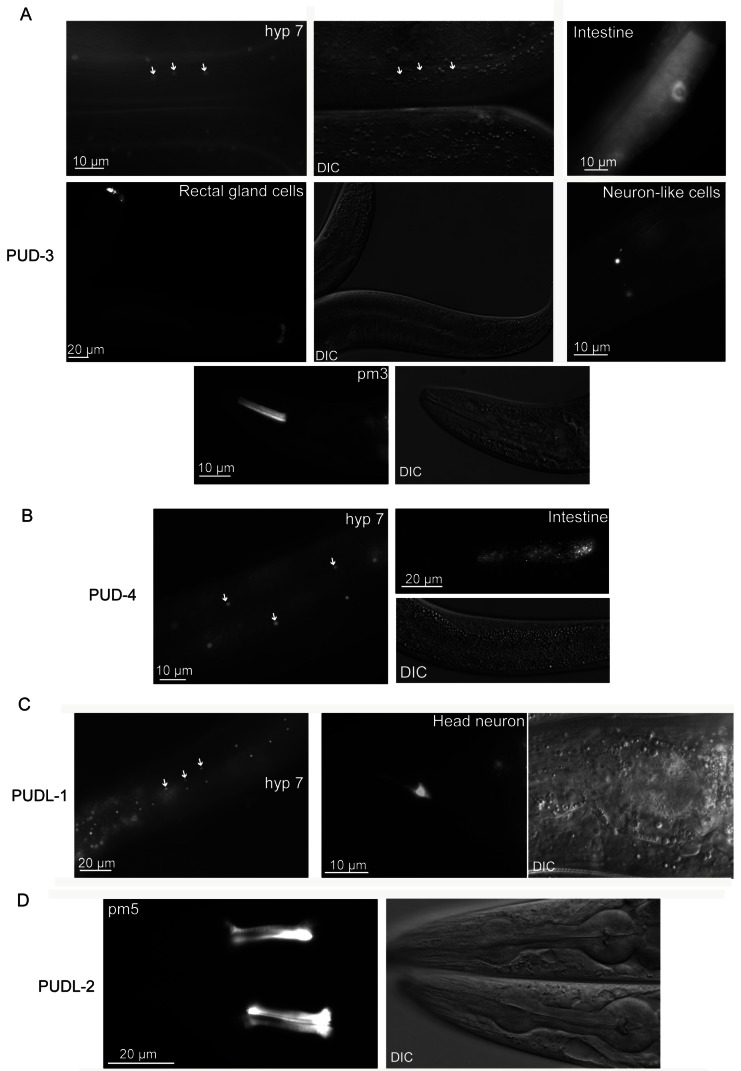
Expression patterns of the paralogs of PUD-1 and PUD-2 in *C. elegans.* (**A**-D) Distributions of the PUD-3, PUD-4, PUDL-1, and PUDL-2 translational GFP fusion proteins, respectively.

The *P*_*pud-3*_ *::pud-3::GFP* transgene was expressed in the nuclei of hyp7, the largest hypodermal cell that wraps around most of the worm body. It was also expressed in the pharyngeal muscle pm3, rectal gland cells, and less frequently, in the intestine and a few neuron-like cells in the head (Figure 6A).

The expression of *P*_*pud-4*_ *::pud-4::GFP* was detected most strongly in the hyp7 nuclei and sporadically in the intestine (Figure 6B).

Also concentrated in the hyp7 nuclei were PUDL-1::GFP and PUDL-2::GFP. Elsewhere, PUDL-1::GFP was seen in a head neuron and PUDL-2::GFP in the pharyngeal muscle pm5 (Figure 6C-D).

Judging by GFP intensity, *pud-1::GFP* and *pud-2::GFP* are the strongest expressers. From mRNA sequencing data of WT day-1 adults (Table S3 in File S1), the sequence reads of *pud-1* and *pud-2* are 10-fold higher than those of *pud-3* and *pud-4*, and about 1000-fold higher than those of *pudl-1* and *pudl-2*. We suspect that *pudl-1* and *pudl-2* are minimally expressed in young adult worms under normal conditions. In contrast, *pud-1* and *pud-2* are constitutively expressed at high levels, and can be induced further. The nuclear localization of the PUD-1/2/3/4 proteins suggests that they might regulate the transcription of the collagen genes whose mRNA levels were altered in the *hq6* mutant (Table 1).

As PUD-1 and PUD-2 form a dimer, we asked whether the rest of the worm homologs are structural counterparts and can also form a dimer. To assess their oligomeric state in solution, we preformed analytical gel filtration experiments (Figure 4C-D). Since these proteins alone have a low solubility, they were analyzed with a fused solubility-enhancing SMT3-tag. Separately, SMT3-PUD-1 and SMT3-PUD-2 eluted in a position corresponding to a monomeric species. Combining them led to the appearance of a new peak corresponding to a dimeric species, which is expected for the PUD-1/PUD-2 heterodimer (Figure 4C). When either SMT3-PUD-1 or SMT3-PUD-2 was mixed with other four homologs, the elution peak stayed the same at the monomeric position (Figure 4D). This indicates that the four homologs cannot form a dimer with either PUD-1 or PUD-2, or with one another. Mixing of all six PUD family proteins led to a dimeric species that can be ascribed to the PUD-1/PUD-2 dimer (Figure 4D). We thus conclude that PUD-3, PUD-4, PUDL-1, and PUDL-2 are monomeric, in contrast with the heterodimeric structure of PUD-1 and PUD-2. The sequence alignment shows that the residues at the dimer interface of PUD-1 and PUD-2 are not well conserved in other homologs (Figure 5E), corroborating the different oligomeric states of these proteins.

## Discussion

There is no doubt that the downstream targets of DAF-16 are responsible for the remarkable longevity of *daf-2*, *age-1*, and other IIS mutants. However, a concrete and comprehensive list of DAF-16 targets has remained elusive despite the efforts of multiple research groups [40]. Yet, even more difficult are the tasks of verifying the functions of candidate targets. Here we find that PUD-1 and PUD-2, two proteins displaying the most notable abundance increase in a quantitative proteomics analysis of *daf-2*, appear to have no contribution to longevity (Figure 2A–D), tolerance of stress (Figure 2E-F), and dauer formation (not shown). This is similar to *sod-3*, which encodes an inducible mitochondrial superoxide dismutase and is a *bona fide* DAF-16 target once believed to be beneficial to longevity. Despite its marked increase of expression in *daf-2* mutants [41], independent reports recently showed that deletion of *sod-3* had no effect on either WT or *daf-2* lifespan [42-44]. In fact, all five *C. elegans sod* genes are dispensable for WT lifespan [45].

IIS controls many biological processes. Some of them are relevant to aging and some probably not. This study and the efforts of others trying to elucidate whether and how some of the DAF-16 targets contribute to longevity have highlighted the complexity of aging regulation. Initially, many of the genes that had been tested because their mRNA levels increased or decreased in *daf*-2(-) vs. *daf*-2(+) backgrounds appeared to contribute to *daf-2* longevity [10]. However, as more genes were examined, it became clear that not all changes in *daf-2* were in the direction of extending lifespan [8]. In fact, some protein abundance changes in *daf-2* such as the increase of ACO-2 may serve to shorten lifespan, possibly as part of a compensation mechanism acting to restore the WT status [8]. *daf-2* mutants are resistant to Aβ toxicity and have increased levels of the PUD-1/PUD-2 heterodimer. Our finding that mutant worms lacking *pud-1* and *pud-2* were more resistant to paralysis caused by Aβ seems to provide another example (Figure 3).

## Supporting Information

File S1 Figure S1., Increase of the PUD-1 and PUD-2 protein abundance in the *daf-2* mutant is mostly due to post-transcriptional regulation independent of *daf-16*. (**a**) Quantitative RT-PCR of *pud-1* and *pud-2* in WT, *daf-16*(*mu86*)*, daf-2*(*e1370*), and *daf-16*(*mu86*)*; daf-2*(*e1370*) worms. Data are shown as mean ± s.e.m. of three independent experiments, each with duplicate measurements. (**b**) Anti-PUD-1 and anti-PUD-2 western blots showing the protein levels in whole-worm lysates of the indicated strains. The anti-tubulin signals control for loading. (**c**) Summary of the densitometry measurements of three independent experiments including the one shown in (**b**), expressed as mean ± standard deviation. * *p* < 0.01 *vs*. the wild type N2. Figure S2. PUD-1::GFP and PUD-2::GFP expressed from transgene arrays under the control of their native promoters. (**a**) GFP expression constructs of PUD-1 (pYG2) and PUD-2 (pWX1). (**b**-**c**) Both are strongly expressed in the intestine and less strongly in the hypodermis. The inset shows that PUD-1::GFP or PUD-2::GFP is expressed in the nucleoplasm of intestinal cells, largely excluded from the nucleolus except for one or more puncta. (**d**-**e**) A temperature shift from 20 °C to 27 °C stimulated the expression of PUD-1::GFP (d) and PUD-2::GFP (**e**). Figure S3. *hqIs28* and *hqIs60*, but not *hqIs24*, extended *daf-2* lifespan. *hqIs28* and *hqIs60* extended the lifespan of *daf-2*(*e1370*) (a) or *daf-2*(*RNAi*) (b) mutants, whereas *hqIs24* (c) did not. Figure S4. The distribution of the PUD gene family members in the *C. elegans* genome and the location of *niDf209*. (**a**) *pud-1.2, pud-2.2, pud-3, pud-4, pud-1.1* and *pud-2.1* are next to each other on Chromosome V. *pud-1.2* is a perfect duplicate of *pud-1.1* and *pud-2.2* is a perfect duplicate of *pud-2.1*. The boundary of *niDf209* as annotated in the wormbase.org is not precise. The actual endpoints of *niDf209* are indicated. The flanking sequences are GTACTGTAGGCC [15979 bp deletion] [1667 bp insertion] TGTAATTCCACG. The 1667 bp insertion consists of a 67-bp sequence“TACTGTAGGATTACTGTAGTTTAAAAAAGGGATTTCAGCTTTCGAAAAGGTATTGAACGAAGATTAG” (indicated by a very short blue segment right before an orange arrow) followed by an inverted duplicate of a 1600-bp fragment from *Y19D10B.5* “TGTAGAATTCT C….AACGGAACGGTT” (orange arrows). (**b**) *pudl-1* and *pudl-2* are adjacent genes on Chromosome IV. Figure S5. Transplanting *niDf209* from JU258 to the N2 background by homologous recombination. Figure S6. pYG17, an RNAi construct for knocking down multiple members of the PUD gene family. Figure S7. Sequence alignment of the PUD gene family proteins. Sequences were aligned by MUSCLE program and slightly adjusted. The abbreviation of species name and Genebank ID are indicated for each sequence. The secondary structures of PUD-2 are indicated above the alignment. Shading of black, grey and light grey represent 100%, 80% and 60% conservation, respectively. Table S1. Data collection and refinement statistics. Table S2. Endogenous promoter-driven expression of GFP translational fusion proteins. Table S3. mRNA levels of *pud* family genes. Table S4. Constructs. Table S5. *C. elegans* Strains.
