# Correction: Characterization of PUD-1 and PUD-2, Two Proteins Up-Regulated in a Long-Lived *daf-2* Mutant

**DOI:** 10.1371/annotation/6b155146-de73-4733-83b0-62224d84717e

**Published:** 2013-12-20

**Authors:** Yue-He Ding, Yun-Guang Du, Shukun Luo, Yu-Xin Li, Tie-Mei Li, Sawako Yoshina, Xing Wang, Karsten Klage, Shohei Mitani, Keqiong Ye, Meng-Qiu Dong

There is an error in the text for the hqEx130 allele, which appears in rows 33- 38 in Table S5. The correction texts for these rows are:

MQD581 hqEx130[pYG35(Ppudl-2::GFP::pudl-2),PL15EK(lin-15)];lin-15B(n765ts) X

MQD693 hqEx285[pYG40(pud-3::GFP::Ppud-4::FLAG::pud-4),PL15EK(lin-15)];lin-15B(n765ts) X

MQD694 hqEx286[pYG40(pud-3::GFP::Ppud-4::FLAG::pud-4),PL15EK(lin-15)];lin-15B(n765ts) X

MQD696 hqEx288[pYG41(pud-4::GFP::Ppud-3::FLAG::pud-3),PL15EK(lin-15)];lin-15B(n765ts) X

MQD700 hqEx292[pYG41(pud-4::GFP::Ppud-3::FLAG::pud-3),PL15EK(lin-15)];lin-15B(n765ts) X

MQD701 hqEx293[pYG41(pud-4::GFP::Ppud-3::FLAG::pud-3),PL15EK(lin-15)];lin-15B(n765ts) X 

