# Supplementary material for: Characterization of PUD-1 and PUD-2, Two Proteins Up-Regulated in a Long-Lived daf-2 Mutant
Source: PLoS One. 2013 Jun 14;8(6):e67158. doi: 10.1371/journal.pone.0067158 (PMC3683130; doi:10.1371/journal.pone.0067158)
Supplement: File S1 — Figure S1., Increase of the PUD-1 and PUD-2 protein abundance in the daf-2 mutant is mostly due to post-transcriptional regulation independent of daf-16. (a) Quantitative RT-PCR of pud-1 and pud-2 in WT, daf-16(mu86), daf-2(e1370), and daf-16(mu86); daf-2(e1370) worms. Data are shown as mean ± s.e.m. of three independent experiments, each with duplicate measurements. (b) Anti-PUD-1 and anti-PUD-2 western blots showing the protein levels in whole-worm lysates of the indicated strains. The anti-tubulin signals control for loading. (c) Summary of the densitometry measurements of three independent experiments including the one shown in (b), expressed as mean ± standard deviation. * p < 0.01 vs. the wild type N2. Figure S2. PUD-1::GFP and PUD-2::GFP expressed from transgene arrays under the control of their native promoters. (a) GFP expression constructs of PUD-1 (pYG2) and PUD-2 (pWX1). (b-c) Both are strongly expressed in the intestine and less strongly in the hypodermis. The inset shows that PUD-1::GFP or PUD-2::GFP is expressed in the nucleoplasm of intestinal cells, largely excluded from the nucleolus except for one or more puncta. (d-e) A temperature shift from 20 °C to 27 °C stimulated the expression of PUD-1::GFP (d) and PUD-2::GFP (e). Figure S3. hqIs28 and hqIs60, but not hqIs24, extended daf-2 lifespan. hqIs28 and hqIs60 extended the lifespan of daf-2(e1370) (a) or daf-2(RNAi) (b) mutants, whereas hqIs24 (c) did not. Figure S4. The distribution of the PUD gene family members in the C. elegans genome and the location of niDf209. (a) pud-1.2, pud-2.2, pud-3, pud-4, pud-1.1 and pud-2.1 are next to each other on Chromosome V. pud-1.2 is a perfect duplicate of pud-1.1 and pud-2.2 is a perfect duplicate of pud-2.1. The boundary of niDf209 as annotated in the wormbase.org is not precise. The actual endpoints of niDf209 are indicated. The flanking sequences are GTACTGTAGGCC [15979 bp deletion] [1667 bp insertion] TGTAATTCCACG. The 1667 bp insertion consists of a 67-bp [file pone.0067158.s001.pdf]

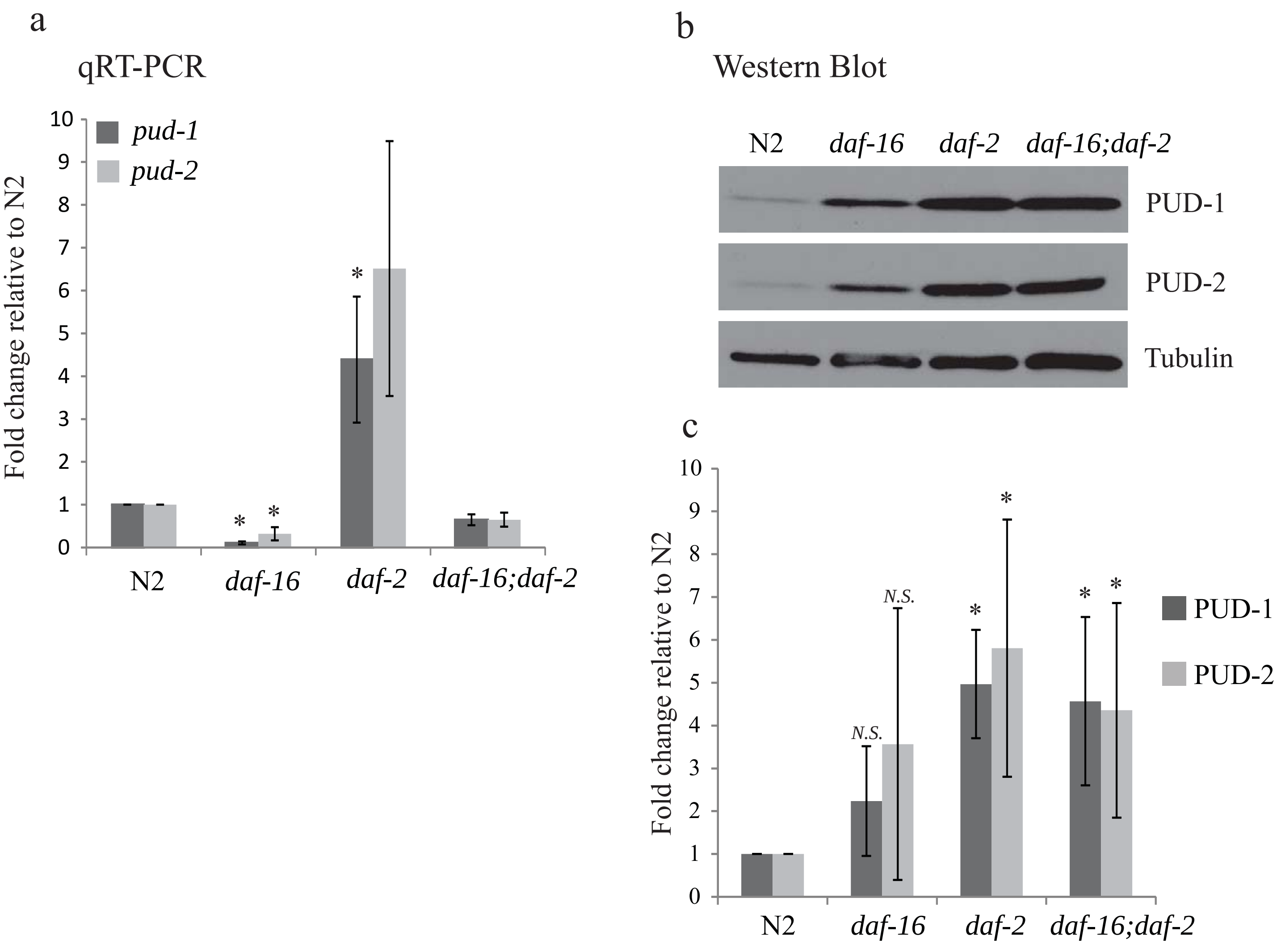
a
qRT-PCR
Fold change relative to N2
10
9
8
7
6
5
4
3
2
1
0
pud-1
pud-2
*
*
*
N2
daf-16
daf-2
daf-16;daf-2
b
Western Blot
N2
daf-16
daf-2
daf-16;daf-2
PUD-1
PUD-2
Tubulin
c
Fold change relative to N2
10
9
8
7
6
5
4
3
2
1
0
N.S.
N.S.
*
*
*
*
PUD-1
PUD-2
N2
daf-16
daf-2
daf-16;daf-2

Figure S1

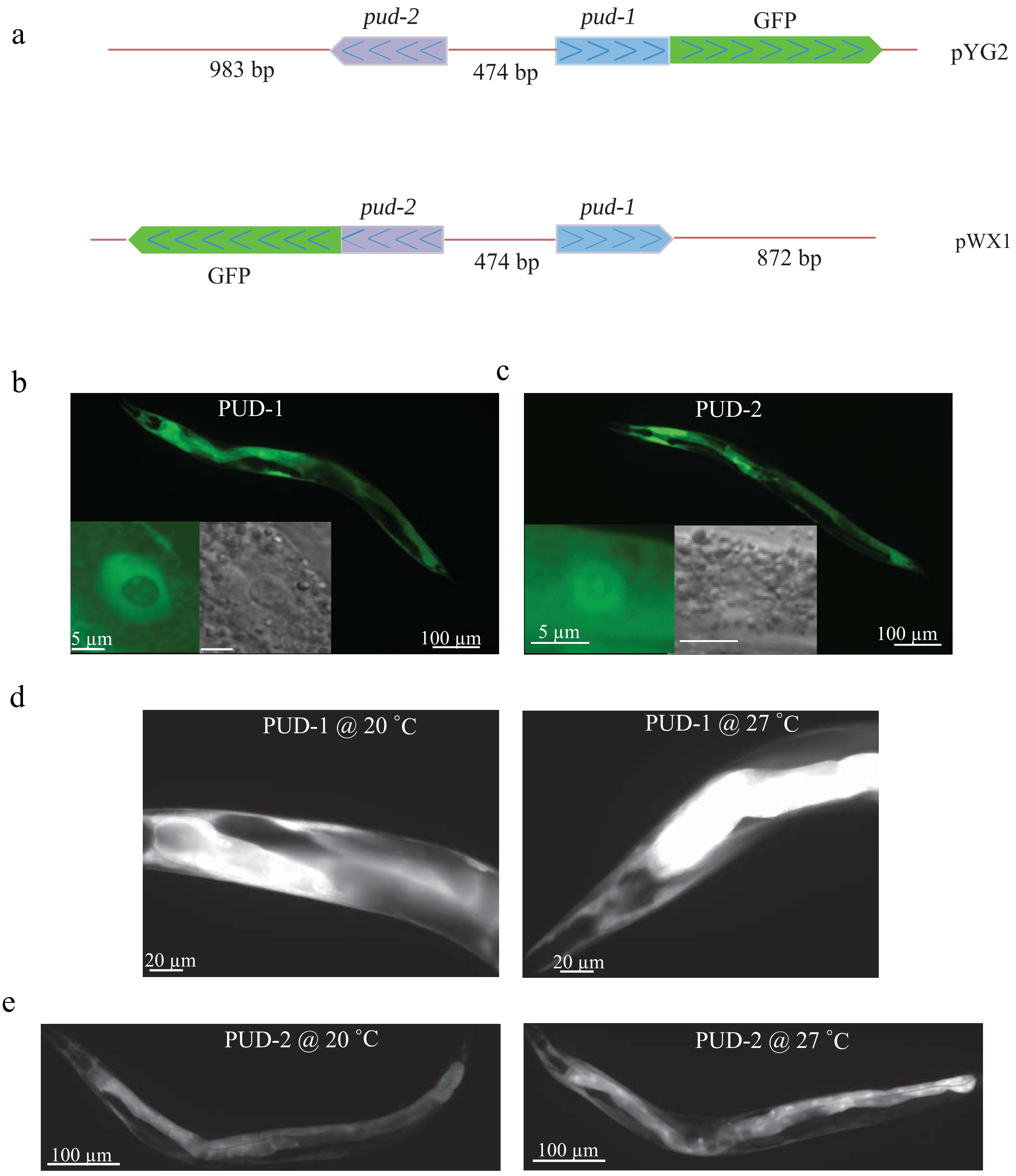
a
pud-2
pud-1
GFP
983 bp
474 bp
pYG2
pud-2
pud-1
474 bp
872 bp
GFP
pWX1
b
PUD-1
5 µm
100 µm
c
PUD-2
5 µm
100 µm
d
PUD-1 @ 20 ˚C
20 µm
PUD-1 @ 27 ˚C
20 µm
e
PUD-2 @ 20 ˚C
100 µm
PUD-2 @ 27 ˚C
100 µm

Figure S2

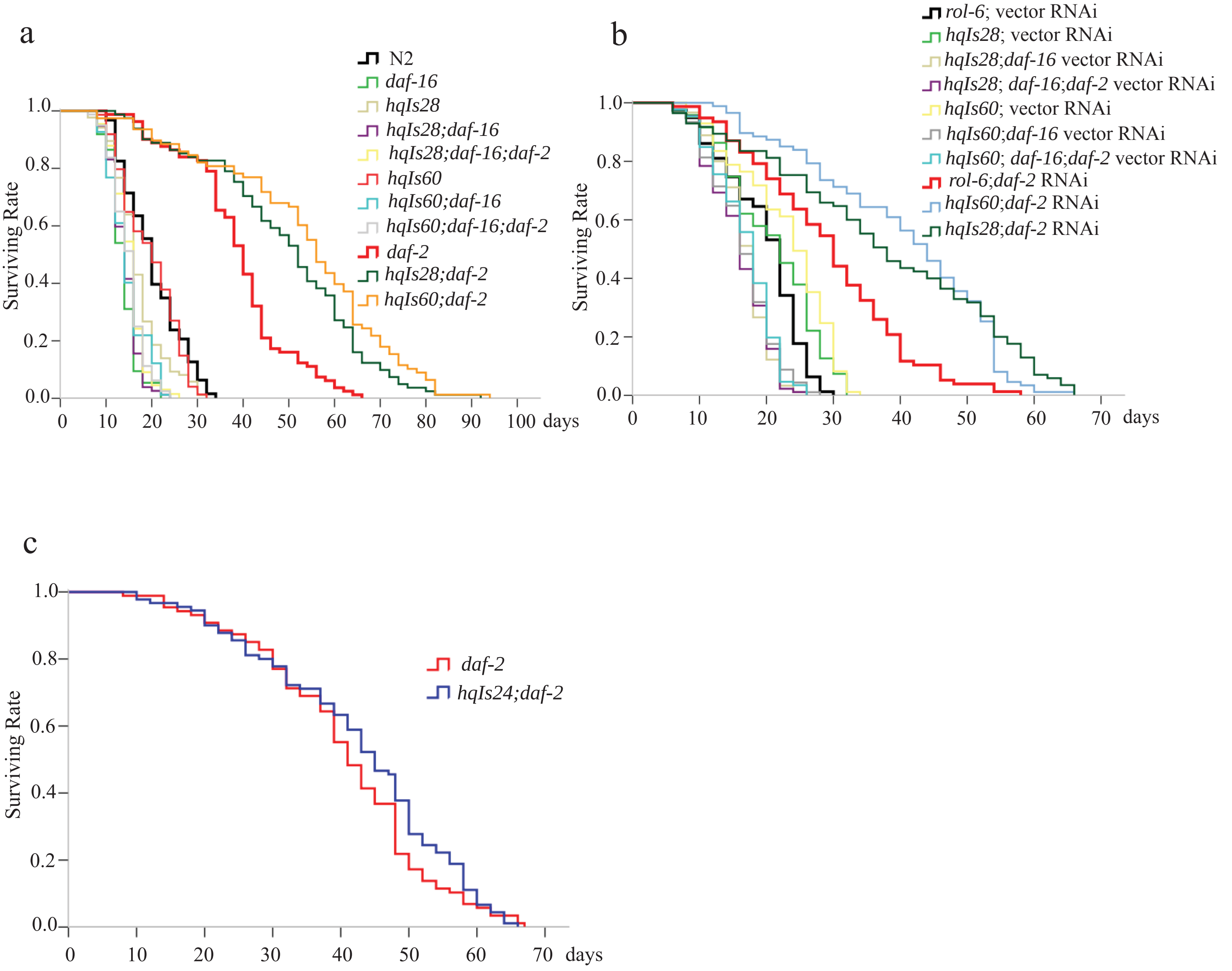
a
N2
daf-16
hqIs28
hqIs28;daf-16
hqIs28;daf-16;daf-2
hqIs60
hqIs60;daf-16
hqIs60;daf-16;daf-2
daf-2
hqIs28;daf-2
hqIs60;daf-2
Surviving Rate
1.0
0.8
0.6
0.4
0.2
0.0
0 10 20 30 40 50 60 70 80 90 100 days
b
rol-6; vector RNAi
hqIs28; vector RNAi
hqIs28;daf-16 vector RNAi
hqIs28; daf-16;daf-2 vector RNAi
hqIs60; vector RNAi
hqIs60;daf-16 vector RNAi
hqIs60; daf-16;daf-2 vector RNAi
rol-6;daf-2 RNAi
hqIs60;daf-2 RNAi
hqIs28;daf-2 RNAi
Surviving Rate
1.0
0.8
0.6
0.4
0.2
0.0
0 10 20 30 40 50 60 70 days
c
daf-2
hqIs24;daf-2
Surviving Rate
1.0
0.8
0.6
0.4
0.2
0.0
0 10 20 30 40 50 60 70 days

Figure S3

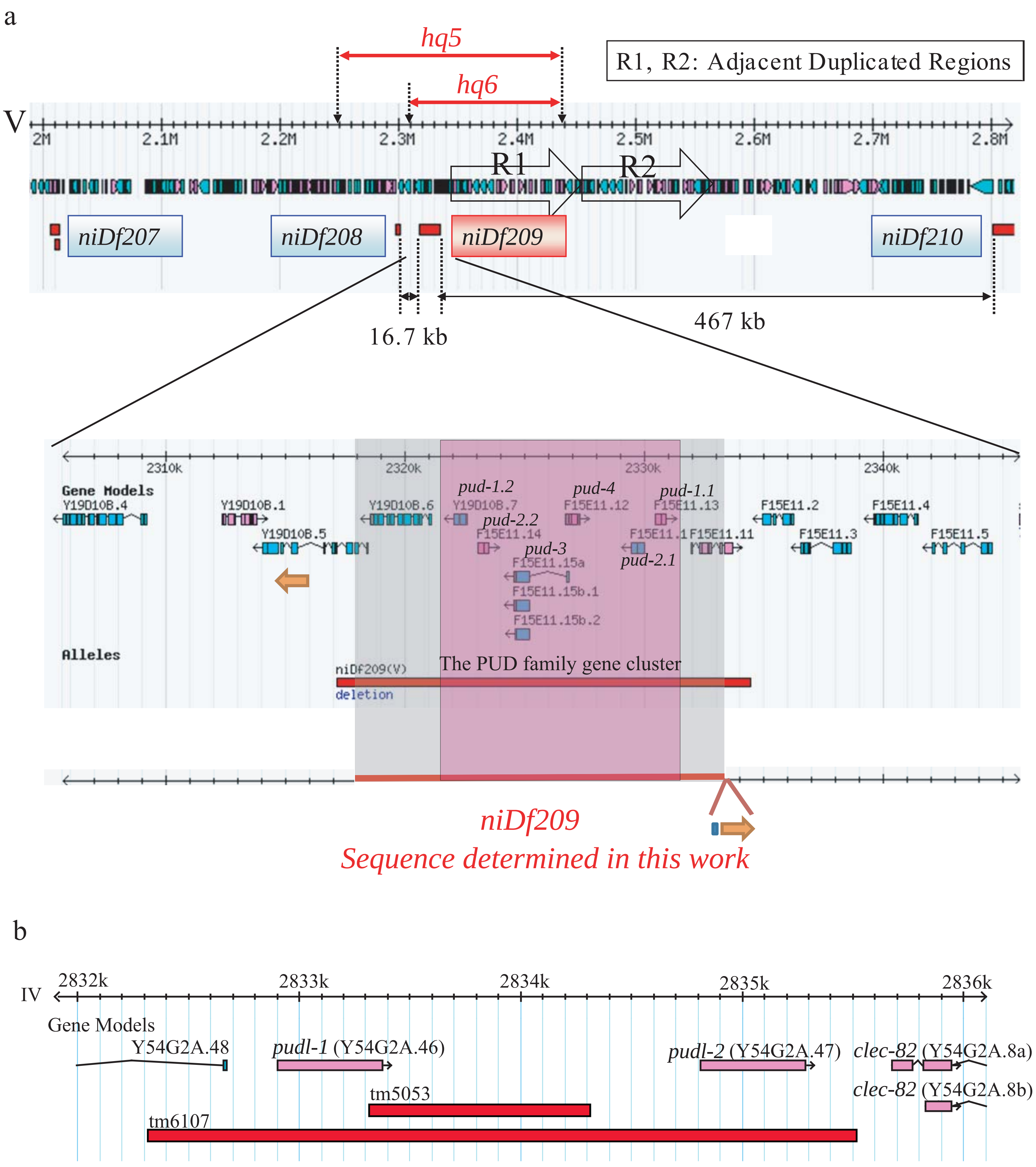
a
hq5
hq6
R1, R2: Adjacent Duplicated Regions
V
2M
2.1M
2.2M
2.3M
2.4M
2.5M
2.6M
2.7M
2.8M
R1
R2
niDf207
niDf208
niDf209
niDf210
16.7 kb
467 kb
2310k
2320k
2330k
2340k
Gene Models
Y19D10B.4
Y19D10B.1
Y19D10B.5
Y19D10B.6
pud-1.2
Y19D10B.7
pud-2.2
F15E11.14
pud-3
F15E11.15a
F15E11.15b.1
F15E11.15b.2
pud-4
F15E11.12
pud-1.1
F15E11.13
F15E11.1
pud-2.1
F15E11.11
F15E11.2
F15E11.3
F15E11.4
F15E11.5
Alleles
niDf209(V)
deletion
The PUD family gene cluster
niDf209
Sequence determined in this work
b
IV
2832k
2833k
2834k
2835k
2836k
Gene Models
Y54G2A.48
pudl-1 (Y54G2A.46)
pudl-2 (Y54G2A.47)
clec-82 (Y54G2A.8a)
clec-82 (Y54G2A.8b)
tm5053
tm6107

Figure S4

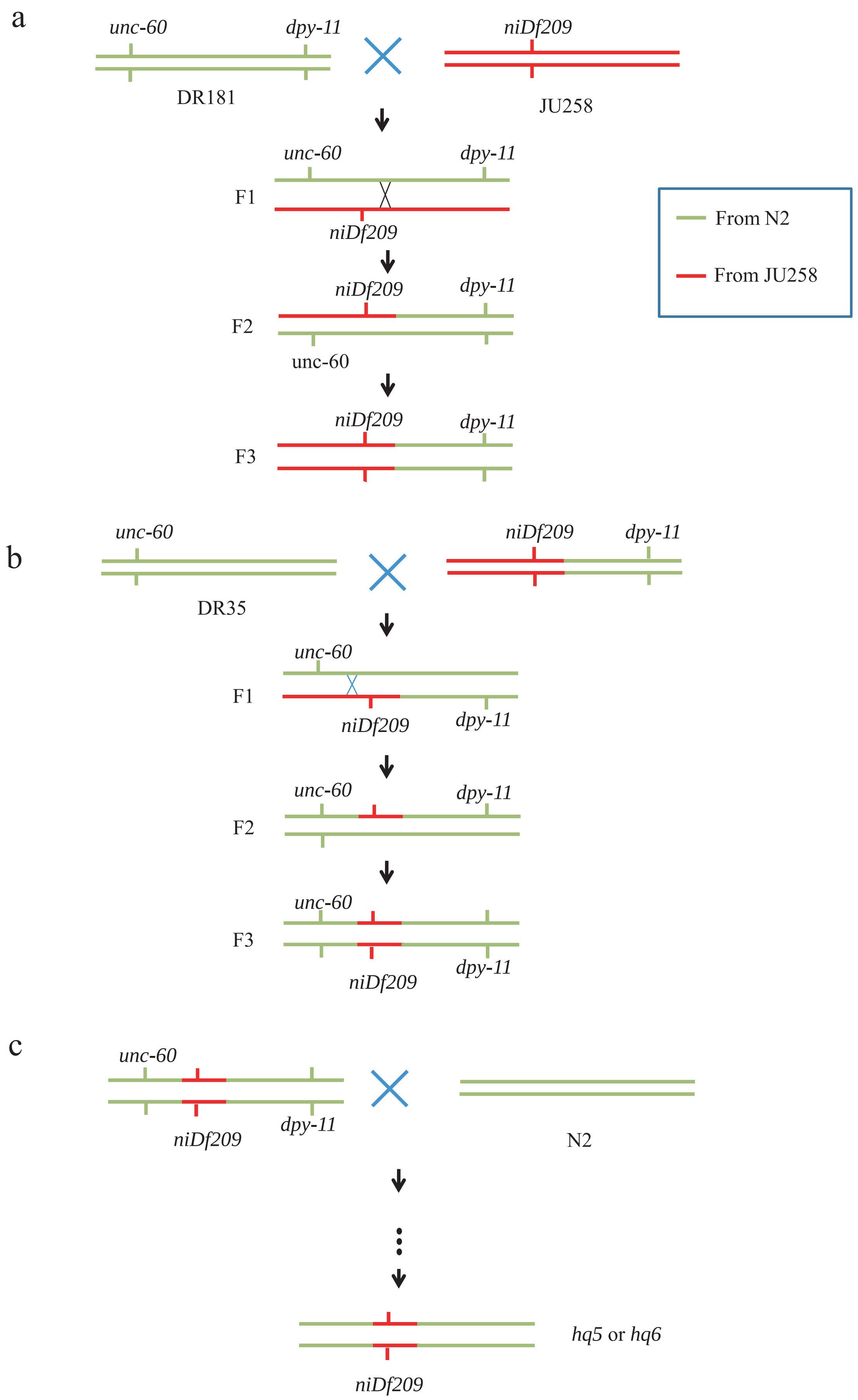
a
unc-60
dpy-11
DR181
niDf209
JU258
F1
unc-60
dpy-11
niDf209
From N2
From JU258
F2
niDf209
dpy-11
unc-60
F3
niDf209
dpy-11
b
unc-60
DR35
niDf209
dpy-11
F1
unc-60
niDf209
dpy-11
F2
unc-60
dpy-11
F3
unc-60
niDf209
dpy-11
c
unc-60
niDf209
dpy-11
N2
hq5 or hq6
niDf209

Figure S5

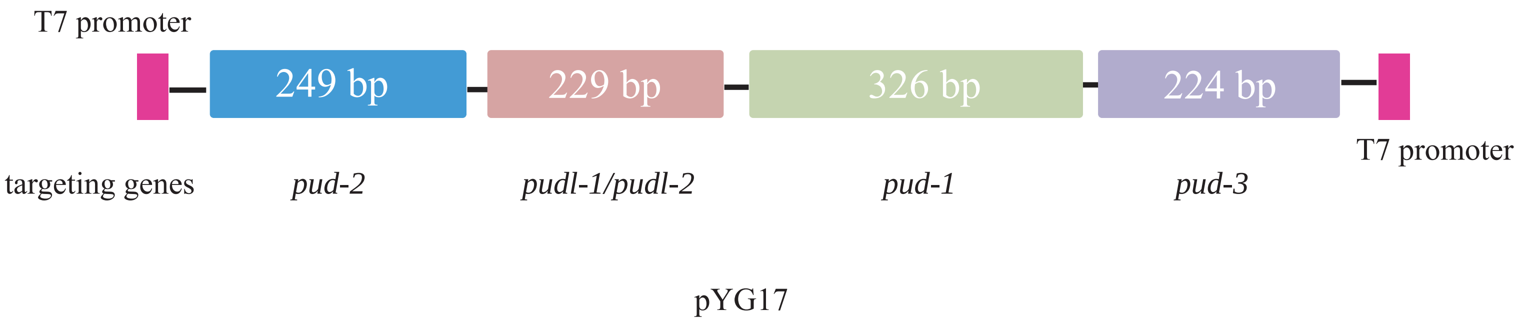
T7 promoter
249 bp
229 bp
326 bp
224 bp
T7 promoter
targeting genes
pud-2
pudl-1/pudl-2
pud-1
pud-3
pYG17

Figure S6

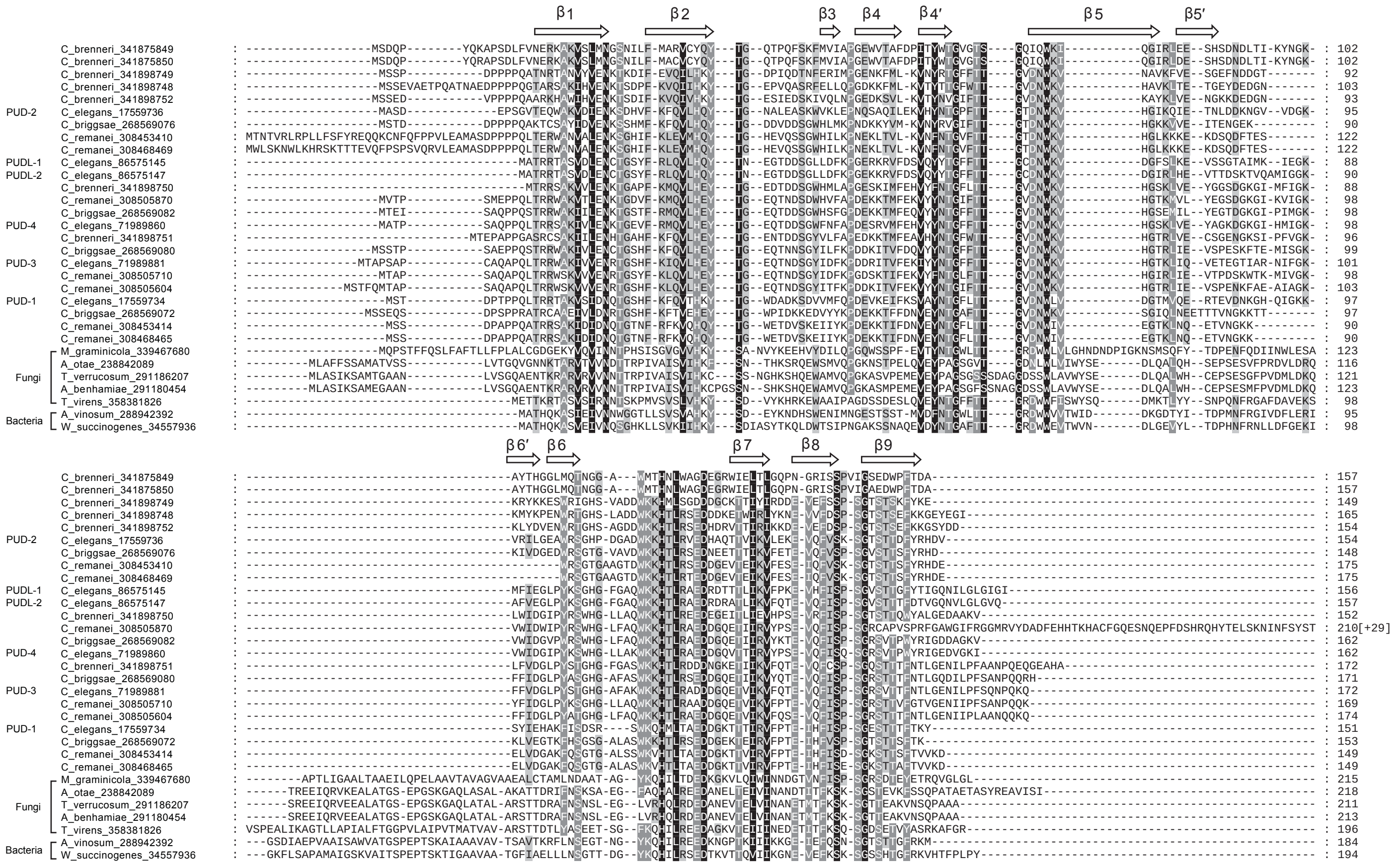
β1
β2
β3
β4
β4′
β5
β5′
β6′
β6
β7
β8
β9
PUD-2
PUDL-1
PUDL-2
PUD-4
PUD-3
PUD-1
Fungi
Bacteria
C_brenneri_341875849
C_brenneri_341875850
C_brenneri_341898749
C_brenneri_341898748
C_brenneri_341898752
C_elegans_17559736
C_briggsae_268569076
C_remanei_308453410
C_remanei_308468469
C_elegans_86575145
C_elegans_86575147
C_brenneri_341898750
C_remanei_308505870
C_briggsae_268569082
C_elegans_71989860
C_brenneri_341898751
C_briggsae_268569080
C_elegans_71989881
C_remanei_308505710
C_remanei_308505604
C_elegans_17559734
C_briggsae_268569072
C_remanei_308453414
C_remanei_308468465
M_graminicola_339467680
A_otae_238842089
T_verrucosum_291186207
A_benhamiae_291180454
T_virens_358381826
A_vinosum_288942392
W_succinogenes_34557936

Figure S7

Table S1. Data collection and refinement statistics

Crystal form	PUD-1/PUD-2 (Se)	PUD-1 (9-151)/PUD-2 (7-152)
**Data collection**		
Space group	$P3_221$	$P2_12_12_1$
Cell dimensions		
a, b, c (Å)	149.49, 149.49, 90.02	52.974, 89.78, 102.50
α, β, γ (º)	90, 90,120	90, 90, 90
Wavelength (Å)	0.97940	0.99985
X-ray source	SSRF	SSRF
Resolution range (Å) [a]	30-3.6(3.66-3.60)	25-1.9(1.93-1.90)
Unique reflections	13879	38790
Redundancy	12.2 (11.3)	4.4(3.1)
I/σ	34.7(3.9)	22.9(2.5)
Completeness (%)	100.0(100.0)	98.9(88.6)
R_{merge} [b]	0.119 (0.740)	0.073(0.446)
**Structure refinement**		
Resolution range (Å)		25-1.90(1.95-1.90)
No. reflections		37022(148)
No. atoms		2665
R_{work} [c]		0.192 (0.262)
R_{free} [d]		0.225 (0.278)
Average B factor ($Å^2$)		31.1
Rmsd bond length (Å)		0.008
Rmsd bond angles (º)		1.212

[a]The values for the data in the highest resolution shell are shown in parentheses.

[b]$R_{merge} = \sum|I_i - I_m|/\sum I_i$, where I_i is the intensity of the measured reflection and I_m is the mean in2tensity of all symmetry related reflections.

[c]$R_{work} = \sum_h|F_o - F_c|/\sum_h F_o$, where F_o and F_c are the observed and calculated structure factor amplitudes of reflection h.

[d]R_{free} is the same as R_{work}, but calculated on 5% reflections not used in refinement

Table S2. Endogenous promoter-driven expression of GFP translational fusion proteins

Protein	Expression pattern in adult *C. elegans*	Note
PUD-1<br>(MosSCI)	all intestinal cells (cytoplasmic and nucleoplasmic)<br>hypodermal cells (cytoplasmic, occasionally nuclear) and appearing to label Fibrous organelles (FOs)	Expression starts before the comma stage in many cells, although the gut cells have the strongest signal.
PUD-2<br>(MosSCI)	all intestinal cells (cytoplasmic and nucleoplasmic)<br>hypodermal cells (cytoplasmic, occasionally nuclear), with very weak signal in FO-like structures.	Expression starts before the comma stage (possibly before gastrulation) in many cells.
PUD-3<br>(transgene array)	Hyp7, most prominently in the nuclei<br>intestinal cells (cytoplasmic and nucleoplasmic).<br>Rectal gland cells<br>Sporadic expression in the alae, pharyngeal muscle pm3, head muscle, or a couple of other cells in the head or tail.	
PUD-4<br>(transgene array)	Hyp7, most prominently in the nuclei<br>Sporadic expression in a few other cells in the head, gut, or tail.	
PUDL-1<br>*(transgene array)*	Hyp7, most prominently in the nuclei<br>a neuron-like cell in the head	
PUDL-2<br>(transgene array)	Hyp7, most prominently in the nuclei<br>Pharyngeal muscle pm5	

Table S3. mRNA levels of *pud* family genes

Genes	CDS(nt)	Experiment 1				Experiment 2				Experiment 3			
		Reads	FPKM	Rank	Percentile	Reads	FPKM	Rank	Percentile	Reads	FPKM	Rank	Percentile
pud-1	456	29127	1.2E+07	379	0.0186	33280	2.8E+07	246	0.0121	31681	1.5E+07	315	0.0154
pud-2	465	81781	3.4E+07	171	0.0084	80301	6.1E+07	135	0.0066	59422	2.8E+07	198	0.0097
pud-3	654/519	6608	3.6E+06	1101	0.0540	8323	8.7E+06	579	0.0284	5974	3.7E+06	1080	0.0530
pud-4	321	2779	1.9E+06	2055	0.1008	3124	4.2E+06	1050	0.0515	2755	2.2E+06	1855	0.0910
pudl-1	474	89	6.9E+04	10676	0.5236	69	1.1E+05	10171	0.4988	41	3.8E+04	11857	0.5815
pudl-2	471	35	2.7E+04	12540	0.6150	28	4.1E+04	12307	0.6036	9	8.3E+03	14578	0.7149
Median		344	8.5E+04	10195	0.5000	218	1.1E+05	10195	0.5000	279	8.2E+04	10195	0.5000
Mean		5126	1.6E+06			2796	2.0E+06			4556	1.6E+06		
Total Reads		104507440				56999137				92900050			

Table S4. Constructs

Plasmid	Usage	Construction Processes
pYG2	express PUD-1::GFP with PUD-2	The PCR products amplified from the N2 genomic DNA were cloned into pPD95.77 using PstI/XbaI or PstI/XmaI restriction sites
pWX1	express PUD-2::GFP with PUD-1	same as above
pDYH39	co-express untagged PUD-1 and PUD-2	A single genomic fragment covering both genes (containing the coding sequences, 3'-UTRs, and the shared promoter region) was inserted into an empty vector pMD19T by T-A ligation
pYG29	express untagged PUD-1	The *pud-1* coding sequence was cloned separately into a modified version of pPD95.77 at the BamHI and EcoRI sites downstream of the *pud-1* promoter and upstream of the *unc-54* 3'-UTR
pYG30	express untagged PUD-2	The *pud-2* coding sequence was cloned separately into a modified version of pPD95.77 at the BamHI and EcoRI sites downstream of the *pud-1* promoter and upstream of the *unc-54* 3'-UTR
pDYH53	express a single copy of GFP::PUD-1	The promoter and coding sequences of *pud-1* were inserted into pPD49.26-N-GFP-1. Then, the expression cassettes (including the promoter, coding sequences of GFP and *pud-1,* and *unc-54* 3'-UTR) were subcloned into CFJ151 (Frøkjær-Jensen et al. 2008) at the SbfI/SpeI sites.
pDYH59	express a single copy of PUD-2::GFP	same as above
pDYH61	express a single copy of FLAG::PUD-1	same as above except that GFP was replaced with FLAG
pYG41	express GFP::PUD-3	The promoter and coding sequences of *pud-3* were inserted into pPD49.26-N-GFP-1
pYG40	express GFP::PUD-4	same as above
pYG34	express GFP::PUDL-1	same as above
pYG35	express GFP::PUDL-2	same as above

Table S5. Strains

Strain	*allele*
Bristol N2	*Wild Type*
CF1038	*daf-16(mu86) I*
CF1041	*daf-2(e1370ts) III*
CL2006	*dvIs2[pCL12(unc-54/human Abeta peptide 1-42 minigene) + pRF4]*
EG4322	*ttTi5605 II;unc-119(ed3) III*
MT8189	*lin-15B(n765) X*
DR35	*unc-60(m35)V*
DR181	*unc-60(m35) dpy-11(e224)V*
MQD58	*daf-16(mu86) I;daf-2(e1370ts) III*
MQD62	*hqIs28[pYG2(pud-2::Ppud-1::pud-1::GFP), pRF4(rol-6)]*
MQD191	*hqIs59[pWX1(pud-1::Ppud-2::pud-2::GFP), pRF4(rol-6)]*
MQD192	*hqIs60[pWX1(pud-1::Ppud-2::pud-2::GFP), pRF4(rol-6)]*
MQD239	*hqEx47[pYG28(Ppud-1::pud-1::GFP), pYG26(Ppud-2::pud-2::Cherry), pRF4(rol-6)]*
MQD249	*hqIs28[pYG2(pud-2::Ppud-1::pud-1::GFP), pRF4(rol-6)]; daf-2(e1370ts) III*
MQD250	*hqIs60[pWX1(pud-1::Ppud-2::pud-2::GFP), pRF4(rol-6)];daf-2(e1370ts) III*
MQD260	*hqIs28[pYG2(pud-2::Ppud-1::pud-1::GFP),pRF4(rol-6)];daf-16(mu86) I;daf-2(e1370ts) III*
MQD261	*hqIs60[pWX1(pud-1::Ppud-2::pud-2::GFP), pRF4(rol-6)];daf-16(mu86) I;daf-2(e1370ts) III*
MQD240	*hqIs28[pYG2(pud-2::Ppud-1::pud-1::GFP),pRF4(rol-6)];daf-16(mu86) I*
MQD254	*hqEx30[pDYH39(pud-1::Ppud-2::pud-2),pL15EK(lin-15)];lin-15B(n765ts) X*
MQD255	*hqEx31[pDYH39(pud-1::Ppud-2::pud-2),pL15EK(lin-15)];lin-15B(n765ts) X*
MQD301	*hqEx50[pYG29(Ppud-1::pud-1),pYG30(Ppud-1::pud-2)]*
MQD303	*hqEx52[pYG29(Ppud-1::pud-1),pYG30(Ppud-1::pud-2)]*
MQD320	*hq5 V*
MQD322	*hq6 V*
MQD393	*hq6 V;daf-2(e1370ts) III*
MQD396	*hqIs91[pDYH53(punc-119::unc-119+Ppud-1::GFP::pud-1)] II*
MQD397	*hqIs92[pDYH59(punc-119::unc-119+Ppud-2::GFP::pud-2)] II*
MQD327	*hqIs93[pDYH61(punc-119::unc-119+Ppud-1::FLAG::pud-1)] II ;unc-119(ed3) III*
MQD570	*dvIs2[pCL12(unc-54/human Abeta peptide 1-42 minigene) + pRF4]; F15(hq6) V*
MQD574	*hqEx123[pYG34(Ppudl-1::GFP::pudl-1),PL15EK(lin-15)];lin-15B(n765ts) X*
MQD575	*hqEx124[pYG34(Ppudl-1::GFP::pudl-1),PL15EK(lin-15)];lin-15B(n765ts) X*
MQD580	*hqEx129[pYG35(Ppudl-2::GFP::pudl-2),PL15EK(lin-15)];lin-15B(n765ts) X*
MQD581	*hqEx130[pYG35(Ppudl-2::GFP::pudl-2),PL15EK(lin-15)];lin-15B(n765ts) X*
MQD693	*hqEx130[pYG40(pud-3::GFP::Ppud-4::FLAG::pud-4),PL15EK(lin-15)];lin-15B(n765ts) X*
MQD694	*hqEx130[pYG40(pud-3::GFP::Ppud-4::FLAG::pud-4),PL15EK(lin-15)];lin-15B(n765ts) X*
MQD696	*hqEx130[pYG41(pud-4::GFP::Ppud-3::FLAG::pud-3),PL15EK(lin-15)];lin-15B(n765ts) X*
MQD700	*hqEx130[pYG41(pud-4::GFP::Ppud-3::FLAG::pud-3),PL15EK(lin-15)];lin-15B(n765ts) X*
MQD701	*hqEx130[pYG41(pud-4::GFP::Ppud-3::FLAG::pud-3),PL15EK(lin-15)];lin-15B(n765ts) X*
MQD935	*dvIs2[pCL12(unc-54/human Abeta peptide 1-42 minigene) + pRF4]; tm5053 IV;F15(hq6) V*
MQD975	*dvIs2[pCL12(unc-54/human Abeta peptide 1-42 minigene) + pRF4]; tm6107 IV;F15(hq6) V*
